# Metabolomic signatures associated with pathological angiogenesis in moyamoya disease

**DOI:** 10.1002/ctm2.1492

**Published:** 2023-11-30

**Authors:** Shihao He, Yanru Wang, Ziqi Liu, Junze Zhang, Xiaokuan Hao, Xilong Wang, Zhenyu Zhou, Rong Wang, Yuanli Zhao

**Affiliations:** ^1^ Department of Neurosurgery Beijing Tiantan Hospital Capital Medical University Beijing China; ^2^ China National Clinical Research Center for Neurological Diseases Beijing China; ^3^ Beijing Institute of Brain Disorders Collaborative Innovation Center for Brain Disorders Capital Medical University Beijing China; ^4^ Department of Neurosurgery Peking Union Medical College Hospital Peking Union Medical College and Chinese Academy of Medical Sciences Beijing China

To the Editor:

Our study proposes specific metabolomic changes and biomarkers that can identify different subtypes of moyamoya disease (MMD) for clinical diagnosis. Moreover, LPC supplementation could inhibit pathological angiogenesis, which might be meaningful for new therapeutic target in MMD.

MMD is an uncommon cerebrovascular condition where the intracranial internal carotid arteries gradually become narrower, frequently leading to the occurrence of stroke.^1^ Currently, there are no diagnostic biomarkers for distinguishing among the different subtypes of MMD. Antiplatelet medications are commonly prescribed to prevent the formation of blood clots in narrowing arteries (a potential cause of ischaemic symptoms) in ischaemic MMD. However, these medications may induce a higher risk of bleeding in haemorrhagic MMD.^2^

Geng et al. have used non‐targeted gas chromatography–mass spectrometry (GC–MS) to investigate serum metabolic biomarkers for MMD, including L‐isoleucine and urea.^3^ Lipidomic analysis has suggested that patients with MMD have decreased serum levels of complex membrane glycosphingolipids.^4^ However, separate subgroup analyses could not be performed due to the small number of identified metabolites, with the small cohort size and lack of additional validation in previous studies. Therefore, we used ultrahigh‐performance liquid chromatography high‐resolution mass spectrometry (UHPLC‐HRMS) untargeted metabolomics analysis to find and identify specific metabolomic changes and biomarkers of different subtypes of MMD. After detailed consultation and physical examination, we collected basic data regarding all patients (Tables S1–S6). The utility of UHPLC‐HRMS untargeted metabolomics analysis^5^ identified 887 and 510 differential metabolite features in positive and negative ion modes with analysis of variance (ANOVA) among ischaemic, haemorrhagic MMD, atherosclerotic stenosis (AS) and health control (HC) groups, respectively.

Untargeted metabolomics analysis revealed differential LPC expression between patients with MMD and HC. Specifically, LPC 16:1‐2 expression was significantly lower in patients with ischaemic MMD than in HC. The area under the receiver operating characteristic curve (AUROC) (.9675) showed that LPC16:1‐2 was a strong candidate biomarker for differentiating patients with ischaemic MMD from HC (Figure 1G). Notably, LPC 16:1‐2 level in the positive ion mode was significantly decreased in ischaemic MMD compared to haemorrhagic MMD (Figure S3, Table S14). Differences in metabolites between other subgroups are shown in Tables S7–S18 and Figures S1 and S2.

**FIGURE 1:**
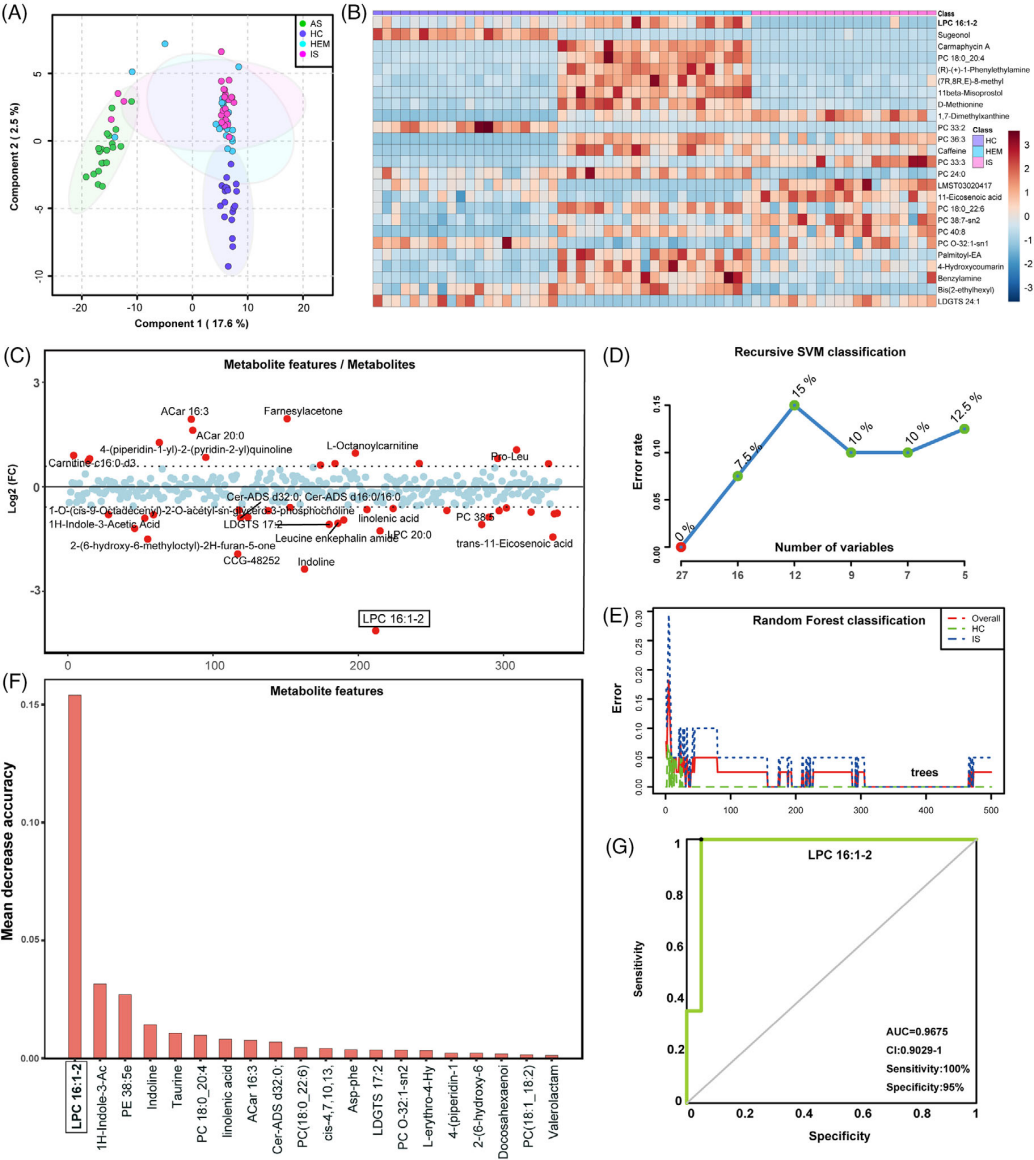
Differential metabolites among the four subgroups. (A) PCA results of the four subgroups in positive ion mode. The ellipses of different colours indicate the 95% confidence interval of the four subgroups. (B) Heatmap of three subgroups in the positive ion mode. The horizontal and vertical axes indicate the sample and metabolite features, respectively. The colour of the dots in the figure represents the intensity of the metabolite levels detected in untargeted metabolomics. (C) The map of metabolite features/metabolites fold change between ischaemic MMD and HC in positive ion mode. |log2 (fold change)| ≥ 1.5 was set as the threshold for significantly differential expression. (D) The results of recursive SVM classification between ischaemic MMD and HC in positive ion mode. The horizontal axis indicates the number of metabolic mass spectrometric features, while the vertical axis indicates the error rate of model prediction. The error rate changed with the number of variables. The error rate decreased to 0 with 27 metabolic features, which indicated great credibility of metabolome results. (E) The results of random forest classification between ischaemic MMD and HC in positive ion mode. The horizontal and vertical axes indicate the number of trees produced with using same number of metabolite features using algorithm and error rate of model, respectively. The green and blue lines represent the healthy control group and ischaemic MMD group, respectively. The red line represents the correlation between the overall error rate and number of trees. It could be seen that error rate significantly decreased to less than .1 near to 0 with increasing of the number of trees, which indicated accuracy of metabolome results. (F) The mean decrease in accuracy plot of the different metabolite features with random forest analysis between ischaemic MMD and HC in the positive ion mode. The average reduction in accuracy represents the model's performance when excluding each individual metabolite. A high value indicates that the metabolite is crucial for the predictive utility of the model. (G) The ROC curve of LPC 16:1‐2. The horizontal axis indicates specificity, while the vertical axis indicates sensitivity.

Moreover, we have whole‐exome sequencing (WES) with blood of the same 53 MMD participants and the same 20 healthy controls in untargeted metabolomics group. We found that RNF213:c.14429G>A, p.R4810K variant existed in 15 MMD patients, and there was no significant correlation between RNF213, p.R4810K variant and LPC levels shown in Table S19.

RNA sequencing (RNA‐seq) analysis of superficial temporal artery (STA) samples revealed that PLA2G2A and PLA1A were down‐expressed significantly in MMD patients compared with controls shown in Figure S4. And PLA2G expression was significantly lower in the middle cerebral artery (MCA) than in the STA in MMD patients as shown in Table S20. Moreover, LPCAT4 and MFSD2A were all up‐expressed in MMD compared with non‐MMD patients as shown in Figure S4. Moreover, we assessed the expression of serum LPC‐related enzymes with ELISA to identify the results of RNA‐seq. And the results of ELISA were consistent with RNA‐seq (Figure S5).

We constructed a cell model of moyamoya disease using serum stimulation to verify the function of the above metabolites.^6^ The apoptotic ratios of HBVSMCs in the ischaemic and haemorrhagic subgroups were significantly lower than HC (Figure 2A,B). Cell viability was significantly increased in three MMD groups. In all the MMD subgroups, monocyte chemotactic protein‐1 (MCP‐1) and NO levels in human brain vascular smooth muscle cells (HBVSMCs) were significantly increased, with the ischaemic subgroup showing the highest level. Levels of reactive oxygen species (ROS) were significantly increased in MMD subgroups. Moreover, the percentage of cells in the S phase was significantly higher in patients with MMD than in HC (Figure 2F,G), indicating increased cell proliferation. Additionally, the percentage of covered area, total tube length and total loops were significantly higher in the MMD groups than in HC (Figure 3 and Figure S8). To identify the effects of LPC level, we constructed cell models with different concentrations of LPC (1, 10, 25, 50 μM). Low LPC concentrations had the similar effects on HBVSMCs and human brain microvascular endothelial cells (HBMECs) to MMD groups shown in Supporting Materials Figures S6 and S7.

**FIGURE 2:**
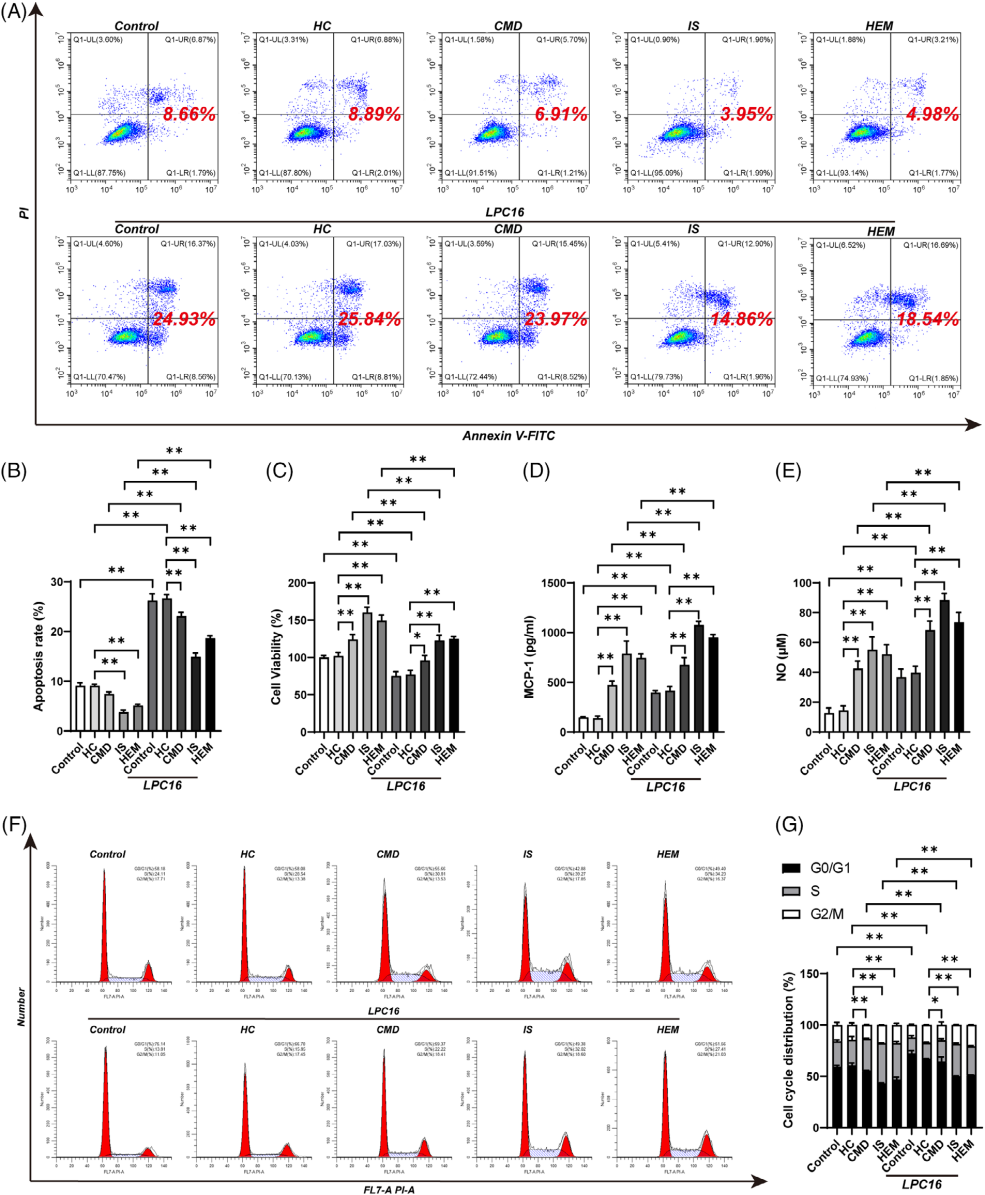
Cell apoptosis, cell viability, MCP‐1 expression, NO expression and cell cycle distribution of HBVSMCs incubated with serum obtained from patients with ischaemic, haemorrhagic and paediatric MMD as well as HC, followed by supplementation with 20 μM LPC 16. (A) The apoptosis ratio of HBVSMCs incubated with serum using flow cytometry. The upper row was not supplemented with LPC 16. The row below was supplemented with LPC 16. (B) The apoptosis ratio of HBVSMCs incubated in serum detected by CCK‐8 assay. The five groups on the right were supplemented with LPC 16. (C) The cell viability of HBVSMCs incubated in serum. The five groups on the right were treated with LPC 16. (D) MCP‐1 levels measured by ELISA in HBVSMCs incubated in serum. The five groups on the right were treated with LPC 16. (E) NO levels measured using the NO assay kit in HBVSMCs incubated in serum. The five groups on the right were treated with LPC 16. (F) Number of HBVSMCs treated with serum in different cell cycle phases. The upper row was not treated with LPC 16. The row below was treated with LPC 16. (G) Cell cycle distribution of HBVSMCs treated with serum. The five groups on the right were treated with LPC 16.

**FIGURE 3:**
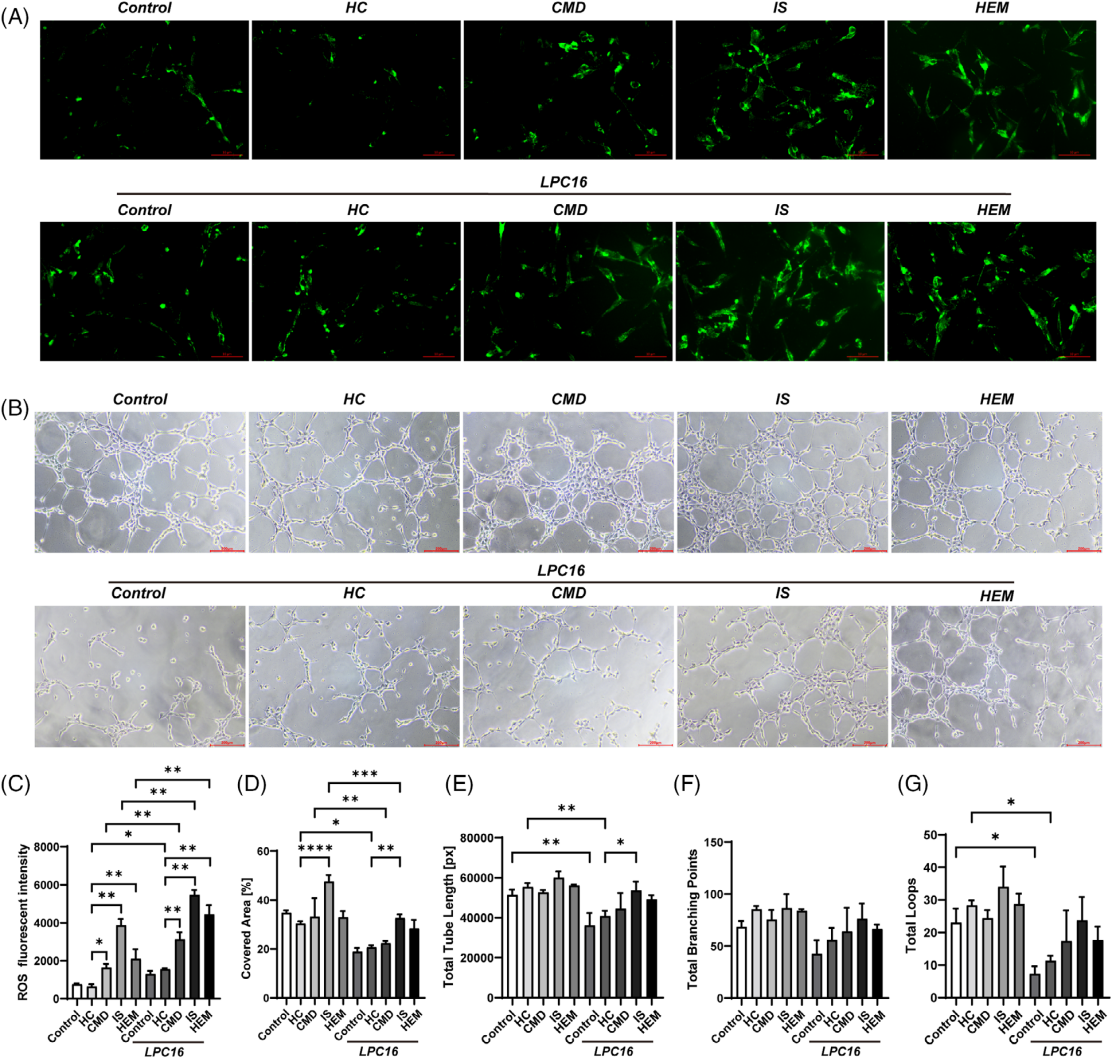
The results of tubule formation assay of HBMECs and ROS production of HBVSMCs incubated with serum obtained from patients with ischaemic, haemorrhagic and paediatric MMD as well as HC, followed by supplementation with 20 μM LPC 16. (A) The fluorescent pictures of ROS level detection assay of HBVSMCs incubated with serum. The upper row was not supplemented with LPC 16. The row below was supplemented with LPC 16. (B) Phase contrast pictures of the tubule formation assay of HBMECs incubated with serum. The upper row was not supplemented with LPC 16. The row below was supplemented with LPC 16. (C) ROS production measured by DCFH‐DA probe in HBVSMCs incubated with serum. The five groups on the right were treated with LPC 16. (D) The percentage of covered area of HBMECs incubated with serum. The five groups on the right were treated with LPC 16. (E) The total tuber length of HBMECs incubated with serum. The five groups on the right were treated with LPC 16. (F) The number of total branching points of HBMECs incubated with serum. The five groups on the right were treated with LPC 16. (G) The number of total loops in the tubule formation assay. The five groups on the right were treated with LPC 16.

Compared with before supplementation, supplementation with LPC 16 (Figure 2C) and LPC 22 (Figures S8–S10) significantly decreased cell viability in all three MMD subgroups. Moreover, supplementation with LPC 16 (in Figure 2D,E) and LPC 22 (Figure S8) significantly increased the MCP‐1, NO levels and ROS levels in the MMD subgroups (Figure 3A,C). Supplementation with LPC 16 significantly decreased the percentage of cells in S phase in all the MMD subgroups (Figure 2F,G), and also significantly decreased covered area, total tube length and total loops in tube formation assay of MMD subgroups (Figure 3 and Figure S9).

LPC, a bioactive lipid generated through pathological processes, is a major constituent of oxidized low‐density lipoprotein, which is known to cause inflammation.^7^ It is suggested to alter the function of cell, including the expression of endothelial adhesion molecules, migration of circulating monocytes, and proliferation and migration of smooth muscle cells, which is involved in cerebral ischaemia and inflammatory diseases.^8^ The sources of LPC in the peripheral circulation include direct absorption from the diet, phosphatidylcholine cleavage by PLA2 in membranes, and hepatic PLA1 activity on PC.^9^ Additionally, LPC can disrupt the integrity of cell membranes and hinder the proper functioning of macromolecules within the membrane, leading to cellular damage in vascular smooth muscle cells.^10^ We found that PLA1 and PLA2 were significantly decreased in MMD, which could lead to low LPC level and decrease its effects.

We present here the most detailed metabolomic data on moyamoya disease to date. LPC 16:1 is downregulated in patients with ischaemic moyamoya disease, suggesting that it may be a candidate biomarker to identify different subtypes of moyamoya disease. LPC supplementation could inhibit abnormal cell viability and cell proliferation of HBVSMCs and angiogenesis function of HBMECs, which may offer a novel therapeutic approach for managing moyamoya disease.

## AUTHOR CONTRIBUTIONS

Not Applicable.

## CONFLICT OF INTEREST STATEMENT

The authors declare they have no conflicts of interest.

## FUNDING INFORMATION

This research was funded by the National Natural Science Foundation of China (Grant Numbers: 81571110 and 81771234 to Yuanli Zhao; grant 82371296 to Rong Wang). These funds covered the expenses related to testing and processing, data collection, analysis and interpretation of the experiment.

## ETHICAL APPROVAL

The study obtained the consent of the participants and parents of children participants in accordance with the Declaration of Helsinki and was approved by the Ethics Committee of the Beijing Tiantan Hospital (KY 2020‐045‐02).

## Supporting information

Supporting Information

## Data Availability

The authors declare that all supporting data are available within the article, the Supporting Materials. WES data have been submitted to Sequence Read Archive (SRA) with the deposited number as PRJNA987311, and the deposited number of RNA‐seq data is PRJNA986272.

## References

[1] Ihara M , Yamamoto Y , Hattori Y , et al. Moyamoya disease: diagnosis and interventions. Lancet Neurol. 2022;21:747‐758. 10.1016/s1474-4422(22)00165-x 35605621

[2] Scott RM , Smith ER . Moyamoya disease and moyamoya syndrome. N Engl J Med. 2009;360:1226‐1237. 10.1056/NEJMra0804622 19297575

[3] Geng C , Cui C , Guo Y , et al. Metabolomic profiling revealed potential biomarkers in patients with moyamoya disease. Front Neurosci. 2020;14:308. 10.3389/fnins.2020.00308 32372905 PMC7186471

[4] Dei Cas M , Carrozzini T , Pollaci G , et al. Plasma lipid profiling contributes to untangle the complexity of moyamoya arteriopathy. Int J Mol Sci. 2021;22:13410. 10.3390/ijms222413410 34948203 PMC8708587

[5] Zheng F , Zhao X , Zeng Z , et al. Development of a plasma pseudotargeted metabolomics method based on ultra‐high‐performance liquid chromatography‐mass spectrometry. Nat Protoc. 2020;15:2519‐2537. 10.1038/s41596-020-0341-5 32581297

[6] Rallo MS , Akel O , Gurram A , Sun H . Experimental animal models for moyamoya disease and treatment: a pathogenesis‐oriented scoping review. Neurosurgical Focus. 2021;51:E5. 10.3171/2021.6.Focus21284 34469865

[7] Zhou Z , Subramanian P , Sevilmis G , et al. Lipoprotein‐derived lysophosphatidic acid promotes atherosclerosis by releasing CXCL1 from the endothelium. Cell Metab. 2011;13:592‐600. 10.1016/j.cmet.2011.02.016 21531341

[8] Bao L , Qi J , Wang YW , et al. The atherogenic actions of LPC on vascular smooth muscle cells and its LPA receptor mediated mechanism. Biochem Biophys Res Commun. 2018;503:1911‐1918. 10.1016/j.bbrc.2018.07.135 30064908

[9] Scagnelli GP , Cooper PS , VandenBroek JM , Berman WF , Schwartz CC . Plasma 1‐palmitoyl‐2‐linoleoyl phosphatidylcholine. Evidence for extensive phospholipase A1 hydrolysis and hepatic metabolism of the products. J Biol Chem. 1991;266:18002‐18011. 1917938

[10] Hsieh CC , Yen MH , Liu HW , Lau YT . Lysophosphatidylcholine induces apoptotic and non‐apoptotic death in vascular smooth muscle cells: in comparison with oxidized LDL. Atherosclerosis. 2000;151:481‐491. 10.1016/s0021-9150(00)00453-6 10924725

