# Supplementary material for: Metabolomic signatures associated with pathological angiogenesis in moyamoya disease
Source: Clin Transl Med. 2023 Nov 30;13(12):e1492. doi: 10.1002/ctm2.1492 (PMC10689969; doi:10.1002/ctm2.1492)
Supplement: Supplementary file 1 — Supporting Information [file CTM2-13-e1492-s001.docx]

**Supporting information**

**Metabolomic signatures associated with pathological angiogenesis**

**in moyamoya disease**

Shihao He^#^,^1,2^ MD, PhD; Yanru Wang^#^, ^1^ MD; Ziqi Liu^#^,^1^ MD, PhD; Junze Zhang,^1^ MD, PhD; Xiaokuan Hao, ^1^ MD; Xilong Wang,^1^ MD; Zhenyu Zhou,^1^ MD; Rong Wang^*1, 2, 3^ MD, PhD; Yuanli Zhao^*1, 2, 3, 4^ MD, PhD

^1^Department of Neurosurgery, Beijing Tiantan Hospital, Capital Medical University, Beijing 100070, China

^2^China National Clinical Research Center for Neurological Diseases, Beijing 100070, China

^3^Center of Stroke, Beijing Institute for Brain Disorders, Beijing 100069, China

^4^Beijing Institute of Brain Disorders, Collaborative Innovation Center for Brain Disorders, Capital Medical University, Beijing, 100069, China

#These authors contributed equally to this work.

***Corresponding authors:**

Rong Wang; E-mail: ronger090614@126.com, Telephone: +86 13001190333

Yuanli Zhao; E-mail: [zhaoyuanli@126.com](mailto:zhaoyuanli@126.com), Telephone: +86 13801121203

**Materials and methods**

**Study approval**

The study obtained the consent of the participants and parents of children participants in accordance with the Declaration of Helsinki and was approved by the Ethics Committee of the Beijing Tiantan Hospital (KY 2020-045-02). Since this was an observational study, the patients underwent the diagnostic procedures and continued treatment at Beijing Tiantan Hospital.

**Participants and Sample preparation**

After detailed consultation and physical examination, we collected basic data regarding all patients (Table S1 and Table S2). A total of 159 Chinese and Han individuals underwent digital subtraction angiography (DSA) to check for MMD (arteriographic appearance characterized by stenosis of the distal intracranial internal carotid artery, extending to the proximal anterior and middle arteries with an extensive collateral network at the base of the brain along with the classic “puff of smoke” appearance)^1^ and 20 atherosclerotic stenosis (AS) patients with ischemic symptoms underwent carotid endarterectomy (ultrasonography detection with a carotid bruit and underwent DSA with percentage of stenosis over 70%^2^)enrolled in this study were also checked with DSA at the Department of Neurosurgery, Beijing Tiantan Hospital, Capital Medical University from July 1, 2021, to December 31, 2022. We performed detailed consultations and physical examinations on patients with MMD to ensure that they did not have any underlying diseases, such as hypertension, diabetes, hyperlipidemia, hyperthyroidism, and surgical history, that could affect the results of this study. Table S2 shows their clinical and demographic characteristics. Written informed consent was obtained from all participants. The study obtained the consent of the participants in accordance with the Declaration of Helsinki and was approved by the Ethics Committee of the Beijing Tiantan Hospital (KY 2020-045-02). Since this was an observational study, the patients underwent the diagnostic procedures and continued treatment at Beijing Tiantan Hospital. We anonymized the data to protect the personal identities of the participants. Additionally, we recruited 30 healthy controls (HC; age range: 18­–45 years). Healthy controls were general of good health who underwent routine checkups and none of these participants or their immediate family has a history of MMD based on interviews and their medical records.

Blood, and superficial temporal artery (STA) samples were collected from patients admitted to Beijing Tiantan Hospital between January 2020 and January 2023. All patients with MMD were divided into three subgroups according to age and type of clinical presentation as follows: children type (CH), ischemic type (IS), and hemorrhagic type (HEM). Serum samples from 40 patients with MMD (IS, n = 20; HEM, n = 20), 20 patients with AS and 20 HC underwent untargeted metabolomics analysis. Moreover, serum samples from 59 new adult patients with MMD (IS, n = 30; HEM, n = 29), and 30 HC were performed with targeted metabolomics. Additionally, we performed ELISAs using serum samples from 25 patients with MMD, 35 patients from the metabolomic assays (CH, n = 20; IS, n = 20; HEM, n = 20), and 20 HC. We performed RNA-seq of 10MCA and 10 STA specimens resected during bypass surgery of 15 new patients with MMD and 1 MMD patient from the metabolomics assays (IS, n = 12; HEM, n = 4). Moreover, we collected 3 more superficial temporal artery (STA) samples from traumatic brain injury (TBI), intracranial pseudoaneurysm (IPA) and arachnoid cyst (AC) patients during surgery in Beijing Tiantan Hospital as controls to compare with RNAseq results of MMD STA samples. Here, the samples obtained from surgery was transferred to store at -80 ℃ within 2 hours after resection and performed with RNA extraction.^3^ We did whole exome sequencing (WES) using blood of 53 MMD patients and 20 healthy controls of untargeted metabolomics. And we collected 2-ml blood samples from all patients with MMD and centrifuged them at 5,000 rpm for 10 min at room temperature, followed by storage at −80℃ for analysis. Table S3­–S6 show the detailed characteristics of the patients for each assay.

**Untargeted metabolomics**

With preparation of QC samples and IDA analysis of all the serum samples, we extracted metabolite mass spectrometry information and keeping time. We extracted metabolite features using MRM analysis and annotated metabolites features. Moreover, UHPLC-TQ/MS was used to quantitatively analyze the high coverage rate and correct the metabolite signatures. The entire scheme was completed within ≈ 5 days. For more details, refer to the protocol described by Zheng et al.^4^

**Targeted metabolomics**

To confirm the differential compounds identified by the untargeted metabolomics analysis, we used ultra-performance liquid chromatography (UPLC) to detect the targeted metabolites in serum samples. Standard compounds (purity, 95%–99%), including lysophosphatidylcholine (LPC) 16, LPC 18, LPC 19, LPC 20, and LPC 22, were purchased from Sigma-Aldrich (St. Louis, MO, USA) and MedChemExpress (New Jersey, USA). Standard solutions were prepared by dissolving the standard compounds in methanol: dichloromethane (1:1) and diluted to doses ranging from 0.1 to500 ppb. The control sample was 20% (vol/vol) CH_3_OH/H_2_O solution. After the standard solution was thawed on ice and vortexed for 5 s, serum was added together with 800 μl IS extraction solution for protein precipitation. After centrifugation for 10 min at 15,000 g at 4℃, 900 μl of the supernatant was transferred to a new centrifuge tube. The sample was reconstituted by adding 60 μl of 90% (vol/vol) H_2_O/CH_3_OH and centrifuged for 10 min at 15,000 g at 4℃. Finally, we injected 5 μl of the supernatant onto a UPLC with SWITCH mode and performed independent analyses.

**Data Processing and Statistical Analyses**

Untargeted and targeted metabolomics analyses were performed using One-step Metabolomics (Dalian ChemData Solution Information Technology Co. Ltd., China, [www.5omics.com](http://www.5omics.com)). We excluded metabolite features whose proportion of non-missing elements was < 80% in each sample group. Missing data for variables were addressed by instead using the minimum value for the respective variable. Principal component analysis (PCA) was performed to observe the intergroup classification trend and sample data outliers in the untargeted and targeted metabolomics analyses.

Mann-Whitney U tests and unpaired t-tests were used for between-group comparisons in the untargeted and targeted metabolomics, respectively. We performed between-group comparisons of the fold-change (FC) values to measure the relative change in the metabolite concentrations. Hierarchical clustering analyses were performed to examine the correlation between the samples and to plot heat maps. Partial least squares discriminant analysis (PLS-DA) was performed for classification methods in metabolomics with quality control normalization; additionally, the correlation coefficients of variable importance on projection (VIP) values were plotted. A VIP score of > 1.0 is an important threshold for determining the significance of metabolic MS features. Orthogonal PLS-DA (OPLS-DA) was performed as a supplement to the PLS-DA method, with five as the maximum number of latent variables and 95% as the confidence interval. The AUROC and false discovery rate q-values were calculated for each candidate metabolite.

Support vector machine (SVM), which is a supervised machine learning method, was used to classify the data and make model predictions for different variables in the untargeted metabolomics. The AUROC value represents the result of modeling the metabolite features. Random forest machine learning method was used as a supplement for data classification in untargeted metabolomics with 500 trees and seven binary tree variables. Potential singular values were identified for the five most significant samples in each between-group comparison.^5-7^ Metabolic pathway analysis was performed using the Human Metabolome Database to annotate metabolites and facilitate visualization of the metabolic pathways. ANOVA analysis was performed for among-group comparisons in untargeted metabolomics, followed by between-group comparisons of IS, HEM, AS and HC.

**Whole exome sequencing**

The exome sequences were efficiently enriched from 0.4 μg genomic DNA using Agilent SureSelect Human All Exon V6 (Agilent USA, Catalog #: 5190-8864)/Agilent SureSelectXT Mouse All Exon library (Agilent, USA, Catalong #: 5190-4643) according to the manufacturer’s protocol. Firstly, qualified genomic DNA was randomly fragmented to an average size of 180-280bp by Covaris LE220R-plus (Covaris, USA). Remaining overhangs were converted into blunt ends via exonuclease polymerase activities. Secondly, DNA fragments were end repaired and phosphorylated, followed by A-tailing and ligation at the 3’ends with paired-end adapters. DNA fragments with ligated adapter molecules on both ends were selectively enriched in a PCR reaction. After PCR reaction, libraries hybridize with liquid phase with biotin labeled probe, then use magnetic beads with streptomycin to capture the exons of genes. Captured libraries were enriched in a PCR reaction to add index tags to prepare for sequencing. Products were purified using AMPure XP system (Beckman Coulter, Beverly, USA), libraries were analyzed for size distribution by Agilent 5400 system（AATI） (Agilent, USA) and quantified by real-time PCR (Life Technologies, USA) (1.5 nM). The qualified libraries were pooled and sequenced on Illumina platforms with PE150 strategy in Novogene Bioinformatics Technology Co., Ltd (Beijing, China), according to effective library concentration and the data amount required. After data quality control with Q30 selection (the percent of bases with Phred-scaled quality scores greater than 30) and sequence alignment by Burrows Wheeler Aligner (BWA) software (Li et al., 2018) and Sambamba software (Tarasov et al., 2015), we detected the variants and performed variant annotation using CoNIFER software (Krumm N et al., 2012) and ANNOVAR software (Wang et al., 2010) respectively. We did the correlation between *RNF213* variants and LPC levels using independent samples t-test comparing LPC 18:2 level between MMD with *RNF213* variants and without *RNF213* variants. WES data has been submitted to Sequence Read Archive (SRA) with the deposited number as PRJNA987311.

**RNA sequencing**

The kits used for library preparation were NEBNext® Ultra™ RNA Library Prep Kit for Illumina® and adaptor index included P5/P7 (primer for PCR), index (identification for different libraries) and Rd1/Rd2 SP (read1/read2 sequence primer, domain combined with primer). RNA extraction from the MCA and STA samples was performed using the RNAsimple Total RNA kit (TIANGEN Biotech, Beijing, China) following the manufacturer’s instructions. The total RNA amount and integrity were assessed using the RNA Nano 6000 Assay Kit of the Bioanalyzer 2100 system (Agilent Technologies, CA, USA). Total RNA was used for RNA sample preparation. Briefly, mRNA was purified from the total RNA using poly T oligo-attached magnetic beads. Fragmentation was conducted using divalent cations at elevated temperatures in First Strand Synthesis Reaction Buffer (5X). First-strand cDNA was synthesized using random hexamer primers and M-MuLV Reverse Transcriptase, followed by RNA degradation using RNaseH. Second-strand cDNA was synthesized using DNA Polymerase I and dNTPs. The remaining overhangs were converted to blunt ends through exonuclease/polymerase activity. After adenylation of 3’ ends of DNA fragments, adaptors with a hairpin loop structure were ligated to prepare for hybridization. To preferentially select cDNA fragments with a length of 370–420 bp, the library fragments were purified using the AMPure XP system (Beckman Coulter, Beverly, USA). Following PCR amplification, the PCR product was purified using AMPure XP beads; additionally, the library was finally obtained and quantified by qRT-PCR using the effective concentration of the library (> 2 nM). After sequencing using Illumina Nova Seq 6000, image data measured using the high-throughput sequencer were converted into sequence data (reads) through CASAVA base recognition. The image data were converted into sequence data (reads) by CASAVA base recognition. Raw data (raw reads) of fastq format were firstly processed through fastp software. In this step, clean data (clean reads) were obtained by removing reads containing adapter, reads containing N base and low quality reads from raw data. At the same time, Q20, Q30 and GC content the clean data were calculated and all the downstream analyses were based on the clean data with high quality. Differential expression analysis of the two conditions/groups (two biological replicates per condition) was performed using the DESeq2 R package (version 1.20.0) and edgeR R package (version 3.22.5). P-values were adjusted using the Benjamini-Hochberg method. Adjusted P-values ≤ 0.05 and |log_2_ (fold change) | ≥ 1 were set as the threshold for significant differential expression. The Gene Ontology (GO) database (http://david.abcc.ncifcrf.gov/) was used to predict gene function. The Kyoto Encyclopedia of Genes and Genomes (KEGG, http://www.genome.ad.jp/kegg/) was used for gene pathway analysis. RNA seq data has been submitted to Sequence Read Archive (SRA). And the deposited number of RNA-seq data is PRJNA986272.

**ELISA**

Phospholipase A1 (PLA1, Biomatik, Kitchener, Ontario, N2C 1N6, Canada), phospholipase A2 (PLA2, Abcam, Cambridge, UK), lysophosphatidylcholine acyltransferase (LPCAT, Aviva systems biology, San Diego, CA, USA), autotaxin (R&D Systems, Inc., Minneapolis, USA), major facilitator superfamily domain-containing protein 2a (Mfsd2a, Biomatik, Kitchener, Ontario, N2C 1N6, Canada), and monocyte chemoattractant protein 1 (MCP-1, R&D Systems, Inc., Minneapolis, USA) levels were assessed using an ELISA kit following the manufacturer’s instructions.

**Cell culture and cell models**

Human brain vascular SMCs (HBVSMCs) and human brain microvascular endothelial cells (HBMECs) (Science Cell, Sunnyvale, California, USA) were cultured in DMEM containing 25 mM HEPES and L-Glutamine (Gibco) with 2% Fetal Bovine Serum (FBS) (Hyclone, Logan, UT, USA). Cells were cultured at 37℃ and 5% CO_2_. When cells reached 90% confluence, the medium was aspirated and the cells were washed twice with FBS. After removing FBS and adding 2 ml 0.25% trypsin-002% EDTA solution, the cells were microscopically observed for approximately 30 s. When cells turned round, they were collected by adding 2 ml DMEM and gently blowing. The supernatant was removed by aspiration after centrifugation at 8,000 rpm at 4℃ for 5 min, followed by resuspension of the cells in DMEM for culture at 37℃ and 5% CO_2_. The medium was changed every other day.

When cells reached 80% confluence, they were transferred to DMEM containing 2% FBS and cultured at 37℃ and 5% CO2 for 24 h. Subsequently, the cells were separately treated with LPC 16 (1, 10, 25, 50 μM) and LPC 22 (1, 10, 25,50 μM) in DMEM containing 2% FBS for 24 h. Cells in the control group were cultured for 48 h after being transferred. HBVSMCs from each group were used in subsequent Cell Counting Kit-8 (CCK-8) assays, flow cytometry to detect apoptosis and the cell cycle, DCFH-DA probe for detecting ROS levels and detection of NO and MCP-1 levels. HBMECs from each group were used in tubule formation assay.

To construct cell models induced with MMD serum, HBVSMCs and HBMECs were transferred to DMEM containing 2% FBS and cultured at 37℃ and 5% CO_2_ for 24 h. Subsequently, cells were treated with 2.5% heat-inactivated serum obtained from IS, HEM, CH and HC groups of previous untargeted metabolomics respectively in FBS-free DMEM for 24 h.^8^ The detailed serum information was shown in Table S6. Next, these cells were transferred to DMEM containing 2% FBS and cultured for 24 h. Cells in the control group were cultured for 72 h after the transfer. Moreover, some of the serum-treated cells were cultured for 24 h in DMEM containing LPC 16/LPC 22 (20 μM) and 2% FBS. HBVSMCs from each group were used in subsequent CCK-8 assays, flow cytometry to detect apoptosis and the cell cycle, DCFH-DA probe for detecting ROS levels and detection of NO and MCP-1 levels. HBMECs from each group were used in tubule formation assay.

**CCK-8 cell viability assay**

Cells from each group were collected after centrifugation and seeded in 96-well plates with triplicate wells per group. After culturing for 48 h and removing the medium, the cells were washed three times with PBS and incubated with 100 μl DMEM containing 10% CCK-8 for 2 h. The absorbance was measured at 450 nm using a Berthold LB941 microplate multifunctional enzyme plate analyzer (Berthold Company, Bad Wildbad, Germany).

**Apoptosis detection by flow cytometry**

Cells from each group were washed twice with PBS and treated with ethylenediaminetetraacetic acid-free trypsin until loss of cell junctions. After the cells were washed twice with PBS and centrifuged at 8,000 rpm for 5 min, about 10^5^ cells were collected, resuspended in 500 μl staining buffer, and incubated with iodide in the dark at room temperature for 15 min after adding 5 μl Annexin V-FITC and 5 μl propidium. Apoptosis was analyzed using a DXFlex flow cytometer (Beckman Coulter, California, USA) for 1 h.

**Cell cycle detection by** **flow cytometry**

Cells from each group were collected in the logarithmic growth phase and the medium was removed. After the cells were washed twice using 0.5 ml FBS and treated with 2 ml 0.25% EDTA-free trypsin, they were collected and centrifuged at 2,000 rpm, 4℃ for 5 min. After collecting the cells and suspending them at a concentration of 10^6^ cells/ml, the cells were resuspended in 70% (v/v) cold ethanol, fixed, and sealed overnight. Next, the cells were centrifuged at 8,000 rpm for 15 min and washed twice with FBS, followed by resuspension in 0.4 ml PBS and transferred to a new tube. Finally, the cells were stained with 500 μl PI/RNase A staining fluid in the dark in an ice bath for 30 min, followed by analysis of the cell cycle using a DXFlex flow cytometer (Beckman Coulter, Brea, California, USA) after filtration through a 300-well nylon mesh.

**ROS level detection**

An ROS assay kit (Beyotime, Shanghai, China) was used for detection of ROS levels. Cells from each group were collected and resuspended in 10 mM DCFH-DA at a concentration of 10^6^ cells/ml. After the cells were incubated at 37℃ in the dark for 20 min and washed three times using serum-free cell culture medium, they were examined by a laser confocal microscope (Zeiss, Oberkochen, Germany), with excitation and emission wavelengths of 488 nm and 525 nm, respectively.

**Detection of NO levels**

Cells from each group were added to an tube containing 8 μl ZnSO_4_ and 8 μl NaOH, followed by centrifugation at 14,000 rpm for 10 min and transfer of 100 μl supernatant to a new EP tube. Subsequently, 200 μl working reagent (prepared according to the instruction with Reagent A (hydrochloric acid), Reagent B (phosphoric acid and NED)and Reagent C (hydrochloric acid and vanadium trichloride), Reagent A: Reagent B: Reagent C=100:4:100) was added to each EP tube, followed by incubation at 60℃ for 10 min. Following short centrifugation and removement of the little crystal precipitation, 250 μl supernatant was transferred to a 96-well plate and the optical density values were determined at 540 nm using the Berthold LB941 microplate multifunctional enzyme plate analyzer (Berthold Company, Bad Wildbad, Germany).

**Statistical analysis**

All results were presented as the mean values±SD. Group comparisons were performed using one-way ANOVA to verify significant differences among groups and Turkey test as post-hoc analysis to compare differences between two groups in cell assays. All statistical analyses were performed using GraphPad Prism 9 software (version 9.4.0, GraphPad Software, San Diego, CA, USA). Statistical significance was set at * p < 0.05, ** p < 0.01.

**Tubule formation assay**

Matrigel glue was transferred to a 4℃ refrigerator for thawing overnight. All pipetting gun heads were kept at 20℃ for pre-cooling. The head was removed and placed on ice for 30 min before the experiment. Subsequently, 50 µl of melted Matrigel glue was added to each well of the 96-well plate, with slight shaking to avoid bubble formation and incubated at 37℃ for 30–60 min to solidify the Matrigel glue. Cells in each group were routinely digested and centrifuged, resuspended in DMEM, and transferred to a 96-well plate at a concentration of 1.5×10^4^ cells/well without touching the glue surface. Triplicate wellswere used for each sample; furthermore, the 96-well plate was routinely incubated at 37℃ for 6 h. Upon complete formation of the lumen, a 96-well plate was observed under a microscope to capture images. The radiographs were crosschecked by two skilled operators. Each experiment was repeated three times. Angiosys 1.0 (https://www.wimasis.com/en/WimTube; TCS Cellworks, Buckingham, England) was used to analyze the images of each group of cells that formed the branching lumens.

**Data availability**

The authors declare that all supporting data are available within the article, the Supplementary Materials. WES data has been submitted to Sequence Read Archive (SRA) with the deposited number as PRJNA987311, and the deposited number of RNA-seq data is PRJNA986272.

**Results**

**Participant characteristics**

The study sample consisted of 70 ischemic MMD patients (mean age ± standard deviation, 37.86 ± 9.57; 31 men, 39 women), 61 hemorrhagic MMD patients (mean age ± standard deviation, 36.62 ± 9.61; 21 men, 40 women), 28 child patients with MMD (mean age ± standard deviation, 9.36 ± 2.88; 11boys, 17 girls), 20 AS patients (mean age ± standard deviation, 59.15 ± 8.82; 12 men, 8 women), and 15 healthy controls (mean age ± standard deviation, 35.43 ± 10.36; 15 men, 15 women). Demographic information of all participants is shown in the Table S1 and S2. The detailed demographic information in different assays was shown in Table S3-S6. We compared demographic differences using chi-squared test and Fisher’s exact test. Although there were demographic differences of participant ages in assays which included CH (children) or AS group (mainly elder people), after eliminating CH and AS group we found there were no significant differences of demographic characteristics among adult MMD groups and HC (p=0.430 in untargeted metabolomics; p=0.699 in ELISA), which inspired that we compared mainly adult MMD patients with healthy controls to identify the potential metabolomic biomarkers in MMD. The other demographic characteristics showed no significant differences among participants.

**Untargeted metabolic profiles of patients with MMD versus HC**

LPC 18:2 levels in the negative ion mode were significantly lower in patients with ischemic MMD than in HC (Figure S2A, S2C, and Table S8). The PLS-DA results indicated that the differential expression of LPC 18:2 in patients with ischemic MMD was a crucial and valid characteristic (Figure S2B, S2D). The AUC value of LPC 18:2 (0.9372) indicated that it was also an important biomarker for ischemic MMD (Figure S2E). Notably, LPC 18:2 levels in the negative ion mode were significantly decreased in patients with hemorrhagic MMD (Table S10). Untargeted metabolomics analysis revealed significantly decreased LPC 17:0 levels in the positive ion mode in patients with hemorrhagic MMD than in HC (Table S9), which indicated that decreased serum LPC levels is common in MMD.

LPC 22:6 levels in the negative ion mode were significantly increased in ischemic MMD compared to hemorrhagic MMD (Table S14). These results indicate that differential serum LPC levels may be correlated with the symptom type of MMD. The decrease in LPC 16:1-2 levels in ischemic MMD indicates that LPC 16:1-2 might be a candidate biomarker for identifying different subtypes in patients with MMD and informing therapeutic interventions to decrease the risk of vascular accident in the future.

**Elevated LPC level in AS patients**

We added AS group with untargeted metabolomics to find the potential biomarker which could also identify MMD from AS. As we found LPC level significantly decreased in MMD versus HC, LPC 25:2 and LPC 18:2-sn1 increased significantly in AS compared with healthy controls shown in Table S15. Compared with adult MMD patients, LPC 20:5 expressed significantly higher in AS, which showed that LPC level decreased uniquely in MMD patients.

**WES verified no significant correlation of LPC and** ***RNF213* variants**

WES analysis found that *RNF213*: c.14429G>A, p.R4810K, rs112735431 existed in 15 MMD patients (28.3%) of total 53 MMD patients. As the LPC 18:2 showed significant differential expression in all three groups of MMD in untargeted metabolomics, we verified the correlation between LPC 18:2 level of MMD patients and *RNF213* variants as shown in Table S18. independent samples t-test showed that no significant correlation between LPC 18:2 level and *RNF213* (p = 0.341).

Neither study showed that direct association between RNF213 and dyslipidemia, although RNF213 was the major susceptibility gene for MMD. It has been reported that RNF213 accelerate triglyceride accumulation in lipid droplet by eliminating adipose triglyceride lipase from lipid droplet.^9^ Moreover, unbiased analyses revealed RNF213 as an important modulator of lipotoxicity caused by saturated fatty acids through inhibition of stearoyl-CoA desaturase.^10^ In spite of such evidence, the baseline plasmatic levels of total cholesterol, LDL-cholesterol, and triglycerides seem to be all within the normal limits in MMD patients compared with healthy controls.^11^ Therefore, the effect of RNF213 in lipid metabolism was uncertain in MMD patients. As for LPC, the level of LPC was mainly correlated with PC cleavage effects of PLA2 and PLA1. Our results showed that RNF213 might not play major roles in LPC metabolism of MMD patients. Therefore, the effect of RNF213 in lipid metabolism was uncertain in MMD patients. As for LPC, the level of LPC was mainly correlated with PC cleavage effects of PLA2 and PLA1. Our results showed that RNF213 might not play major roles in LPC metabolism of MMD patients.

**RNA-seq identified abnormal expression of LPC-related enzymes**

We further studied occlusion artery and tried to find out abnormal expressed genes in cerebral artery might cause differential metabolites of MMD. RNA-seq analysis of STA samples revealed that PLA2G2A and PLA1A were down expressed significantly in MMD patients compared with controls shown in Figure S4A, B. And PLA2G expression was significantly lower in the MCA than in the STA in MMD patients as shown in Table S20. Moreover, LPCAT4 and MFSD2A were all up expressed in MMD compared with non-MMD patients as shown in Figure S4A, B. Irregular PLA2, PLA1, LPCAT and MFSD2A expression might result in abnormal production and conversion of LPC which might lead to significant decreased level in MMD.

**Irregular expression of LPC-related enzymes**

Inspired with the results of RNA seq, we performed ELISA to verify level of LPC-related enzymes in MMD patients compared with HC which might cause low LPC in MMD. We assessed the expression of serum LPC-related enzymes. Levels of PLA1 and PLA2 were decreased in patients with MMD (Figure 5A–E), indicating decreased production of LPC. Further, LPCAT and autotaxin levels, which are related to the conversion of LPC, were increased in patients with MMD. Moreover, Mfsd2a levels were significantly increased in MMD patients.

**Cell viability increased with low LPC levels**

Incubation of HBVSMCs with LPC 16 (1, 10, 25, 50 μM) or LPC 22 (1, 10, 25, 50 μM) for 24 hours revealed that different LPC levels were correlated with cell apoptosis and cell viability. The apoptotic ratio of HBVSMCs showed a significant positive correlation with LPC 16 or LPC 22 levels (Figure 2A–B). Cell viability was highest at an LPC 16 concentration of 1 μM (Figure 2C), with a subsequent decrease as the concentration increased. Moreover, the cell viability was highest at an LPC 22 concentration of 10 μM, with a subsequent decrease as the concentration increased. MCP-1 and NO levels were positively correlated with LPC 16 and LPC 22 levels (Figure 2D–E). Specifically, when the concentration of LPC was > 10 μM, MCP-1 levels were significantly increased in patients with MMD compared to HC.

Cell cycle analysis of HBVSMCs incubated with different concentrations of LPC 16 or LPC 22 was performed using flow cytometry. The concentration of LPC 16 or 22 was negatively correlated with the proportion of cells in S phase (Figure 2F, G), indicating decreased proliferation of HBVSMCs. At concentrations ≥ 10 μM, the percentage of HBVSMCs in S phase significantly decreased compared with the control.

**Low LPC levels promoted the formation of tubules**

ROS levels were positively correlated with LPC 16 or LPC 22 levels (Figure 4A, C), which remained even at LPC 16 or LPC 22 concentrations > 10 μM. To elucidate the functional role of low LPC levels in angiogenesis, we performed the tubule formation assay on HBMECs treated with LPC 16 (1, 10, 25, 50 μM) or LPC 22 (1, 10, 25, 50 μM) for 24 h. As shown in Figure 4D, S9D, S9E, S9G, the percentage of covered area, total tube length and total loops were significantly higher in the MMD groups than in HC. Although there were no significant differences in 1 μM LPC, high LPC concentration inhibited the formation of branching points. As shown in Figure S7 the tube length was negatively correlated with the concentration of LPC, with significant differences being observed with concentrations ≥ 25 μM.

**Supplementation with LPC weakened the effects of MMD serum on HBVSMCs and HBMECs**

Compared with before supplementation, supplementation with LPC 16 (Figure 3C) and LPC 22 (Figure S8-S10) significantly decreased cell viability in all three MMD subgroups. Moreover, supplementation with LPC 16 (in Figure 3D, E) and LPC 22 (Figure S8) significantly increased the MCP-1, NO levels, and ROS levels in the MMD subgroups (Figure 4A, C). Supplementation with LPC 16 significantly decreased the percentage of cells in S phase in all the MMD subgroups (Figure 3F, G) and also significantly decreased covered area, total tube length and total loops in tube formation assay of MMD subgroups. (Figure 4, S9).

LPC supplement increased significantly the apoptosis of HBVSMCs in MMD groups and HC compared with before. The effects of LPC on HBVSMCs were reported with similar results in previous studies. It was found that LPC exhibited a dose-dependent cytotoxic effect in VSMCs^12^ and the mechanism responsible for the cytotoxicity might be detergent-like properties of LPC and its impairment on the membrane structure and function.^13^ Our studies showed similar results correlated with previous studies and we thought LPC supplement which lead to significant increase in HBVSMCs in our studies might correlate with the dose of supplement (20 μM) which might cause HBVSMCs apoptosis. Therefore, we thought the mechanism of LPC effects on HBVSMCs needs more studies and the dose-dependent cytotoxic effect should be more clarified to control the dose to decrease the side effects of LPC on HBVSMCs.

_­­_

**Table S1: Clinical characteristics and sample information of MMD patients.**

| No. of Patients | Groups | | Sex | | Age (years) | Hypertension | Diabetes | Coronary heart disease | | Hyperlipidemia | Smoking history (years) | | Alcohol taking (years) | | | Duration of symptoms (months) | Suzuki stage | Usage (Sample) |
| --- | --- | --- | --- | --- | --- | --- | --- | --- | --- | --- | --- | --- | --- | --- | --- | --- | --- | --- |
| P1 | IS | | M | 24 | | NO | NO | NO | | NO | NO | NO | | | 13 | | L3/R1 | targeted metabolomics (Serum) |
| P2 | IS | | F | 23 | | NO | NO | NO | | NO | NO | NO | | | 24 | | L3/R3 | targeted metabolomics (Serum) |
| P3 | IS | | F | 22 | | NO | NO | NO | | NO | NO | NO | | | 4 | | L3/R4 | targeted metabolomics (Serum) |
| P4 | IS | | M | 41 | | NO | NO | NO | | NO | NO | NO | | | 8 | | L5/R4 | targeted metabolomics (Serum) |
| P5 | IS | | F | 39 | | NO | NO | NO | | NO | NO | NO | | | 6 | | L1/R3 | targeted metabolomics (Serum) |
| P6 | IS | | F | 51 | | NO | NO | NO | | NO | NO | NO | | | 6 | | L3/R3 | targeted metabolomics (Serum) |
| P7 | IS | | F | 56 | | NO | NO | NO | | NO | NO | NO | | | 8 | | L2/R3 | targeted metabolomics (Serum) |
| P8 | IS | | F | 49 | | NO | NO | NO | | NO | NO | NO | | | 5 | | L1/R3 | targeted metabolomics (Serum) |
| P9 | IS | | M | 35 | | NO | NO | NO | | NO | 2 | NO | | | 3 | | L5/R1 | targeted metabolomics (Serum) |
| P10 | IS | | M | 45 | | NO | NO | NO | | NO | NO | NO | | | 9 | | L3/R2 | targeted metabolomics, ELISA (Serum) |
| P11 | IS | | F | 56 | | NO | NO | NO | | NO | NO | NO | | | 12 | | L4/R3 | targeted metabolomics (Serum) |
| P12 | IS | | F | 32 | | NO | NO | NO | | NO | NO | NO | | | 4 | | L3/R1 | targeted metabolomics (Serum) |
| P13 | IS | | F | 55 | | NO | NO | NO | | NO | NO | NO | | | 3 | | L3/R5 | targeted metabolomics (Serum) |
| P14 | IS | | F | 32 | | NO | NO | NO | | NO | NO | NO | | | 5 | | L3/R3 | targeted metabolomics (Serum) |
| P15 | IS | | F | 29 | | NO | NO | NO | | NO | NO | NO | | | 6 | | L5/R3 | targeted metabolomics (Serum) |
| P16 | IS | | F | 44 | | NO | NO | NO | | NO | NO | NO | | | 12 | | L4/R5 | targeted metabolomics (Serum) |
| P17 | IS | | M | 36 | | NO | NO | NO | | NO | NO | NO | | | 12 | | L4/R1 | targeted metabolomics (Serum) |
| P18 | IS | | F | 34 | | NO | NO | NO | | NO | NO | NO | | | 6 | | L3/R3 | targeted metabolomics (Serum) |
| P19 | IS | | M | 36 | | NO | NO | NO | | NO | NO | NO | | | 2 | | L4/R2 | targeted metabolomics (Serum) |
| P20 | IS | | F | 25 | | NO | NO | NO | | NO | NO | NO | | | 12 | | L3/R2 | targeted metabolomics (Serum) |
| P21 | IS | | M | 34 | | NO | NO | NO | | NO | NO | NO | | | 36 | | L4/R5 | targeted metabolomics (Serum) |
| P22 | IS | | F | 46 | | NO | NO | NO | | NO | NO | NO | | | 24 | | L1/R2 | targeted metabolomics (Serum) |
| P23 | IS | | F | 39 | | NO | NO | NO | | NO | NO | NO | | | 5 | | L2/R3 | targeted metabolomics (Serum) |
| P24 | IS | | M | 26 | | NO | NO | NO | | NO | NO | NO | | | 4 | | L3/R2 | targeted metabolomics (Serum) |
| P25 | IS | | F | 19 | | NO | NO | NO | | NO | NO | NO | | | 6 | | L1/R2 | targeted metabolomics (Serum) |
| P26 | IS | | F | 43 | | NO | NO | NO | | NO | NO | NO | | | 5 | | L3/R3 | targeted metabolomics (Serum) |
| P27 | IS | | F | 18 | | NO | NO | NO | | NO | NO | NO | | | 4 | | L3/R1 | targeted metabolomics (Serum) |
| P28 | IS | | F | 43 | | NO | NO | NO | | NO | NO | NO | | | 12 | | L3/R1 | targeted metabolomics (Serum) |
| P29 | IS | | F | 51 | | NO | NO | NO | | NO | NO | NO | | | 24 | | L2/R2 | targeted metabolomics, ELISA (Serum) |
| P30 | IS | | F | 45 | | NO | NO | NO | | NO | NO | NO | | | 2 | | L3/R3 | targeted metabolomics (Serum) |
| P31 | HEM | | F | 47 | | NO | NO | NO | | NO | NO | NO | | | 12 | | L2/R4 | targeted metabolomics (Serum) |
| P32 | HEM | | F | 29 | | NO | NO | NO | | NO | NO | NO | | | 4 | | L1/R1 | targeted metabolomics (Serum) |
| P33 | HEM | | F | 42 | | NO | NO | NO | | NO | NO | NO | | | 4 | | L2/R1 | untargeted metabolomics, ELISA (Serum), WES (Blood) |
| P34 | HEM | | F | 45 | | NO | NO | NO | | NO | NO | NO | | | 8 | | L4/R3 | targeted metabolomics (Serum) |
| P35 | HEM | | F | 28 | | NO | NO | NO | | NO | NO | NO | | | 6 | | L5/R4 | targeted metabolomics (Serum) |
| P36 | HEM | | F | 40 | | NO | NO | NO | | NO | NO | NO | | | 12 | | L3/R6 | targeted metabolomics (Serum) |
| P37 | HEM | | F | 41 | | NO | NO | NO | | NO | NO | NO | | | 5 | | L3/R3 | targeted metabolomics (Serum) |
| P38 | HEM | | F | 50 | | NO | NO | NO | | NO | NO | NO | | | 2 | | L2/R3 | targeted metabolomics, ELISA (Serum) |
| P39 | HEM | | F | 50 | | NO | NO | NO | | NO | NO | NO | | | 5 | | L5/R1 | targeted metabolomics (Serum) |
| P40 | HEM | | F | 34 | | NO | NO | NO | | NO | NO | NO | | | 12 | | L3/R2 | targeted metabolomics (Serum) |
| P41 | HEM | | F | 33 | | NO | NO | NO | | NO | NO | NO | | | 6 | | L3/R5 | targeted metabolomics (Serum) |
| P42 | HEM | | F | 33 | | NO | NO | NO | | NO | NO | NO | | | 3 | | L2/R4 | targeted metabolomics (Serum) |
| P43 | HEM | | M | 34 | | NO | NO | NO | | NO | 2 | NO | | | 24 | | L1/R2 | targeted metabolomics (Serum) |
| P44 | HEM | | M | 49 | | NO | NO | NO | | NO | NO | NO | | | 6 | | L2/R3 | targeted metabolomics (Serum) |
| P45 | HEM | | F | 26 | | NO | NO | NO | | NO | NO | NO | | | 12 | | L3/R3 | targeted metabolomics (Serum) |
| P46 | HEM | | F | 26 | | NO | NO | NO | | NO | NO | NO | | | 5 | | L4/R2 | targeted metabolomics (Serum) |
| P47 | HEM | | F | 48 | | NO | NO | NO | | NO | NO | NO | | | 2 | | L2/R3 | targeted metabolomics (Serum) |
| P48 | HEM | | F | 34 | | NO | NO | NO | | NO | NO | NO | | | 24 | | L5/R2 | targeted metabolomics (Serum) |
| P49 | HEM | | M | 39 | | NO | NO | NO | | NO | NO | NO | | | 6 | | L5/R3 | targeted metabolomics (Serum) |
| P50 | HEM | | F | 33 | | NO | NO | NO | | NO | NO | NO | | | 72 | | L5/R3 | targeted metabolomics (Serum) |
| P51 | HEM | | F | 29 | | NO | NO | NO | | NO | NO | NO | | | 6 | | L2/R3 | targeted metabolomics (Serum) |
| P52 | HEM | | M | 20 | | NO | NO | NO | | NO | NO | NO | | | 3 | | L2/R3 | targeted metabolomics (Serum) |
| P53 | HEM | | F | 31 | | NO | NO | NO | | NO | NO | NO | | | 18 | | L2/R2 | targeted metabolomics (Serum) |
| P54 | HEM | | M | 44 | | NO | NO | NO | | NO | NO | NO | | | 4 | | L2/R3 | targeted metabolomics (Serum), RNA seq (MCA, STA) |
| P55 | HEM | | F | 31 | | NO | NO | NO | | NO | NO | NO | | | 2 | | L3/R3 | targeted metabolomics (Serum) |
| P56 | HEM | | F | 28 | | NO | NO | NO | | NO | NO | NO | | | 12 | | L1/R4 | targeted metabolomics (Serum) |
| P57 | HEM | | M | 38 | | NO | NO | NO | | NO | 1 | 1 | | | 7 | | L2/R2 | targeted metabolomics (Serum) |
| P58 | HEM | | F | 28 | | NO | NO | NO | | NO | NO | NO | | | 16 | | L4/R3 | targeted metabolomics (Serum) |
| P59 | HEM | | M | 40 | | NO | NO | NO | | NO | 1 | NO | | | 6 | | L3/R2 | targeted metabolomics (Serum) |
| P60 | HEM | | M | 42 | | NO | NO | NO | | NO | NO | NO | | | 7 | | L1/R3 | targeted metabolomics (Serum) |
| P61 | CH | | M | 4 | | NO | NO | NO | | NO | NO | NO | | | 3 | | L3/R4 | WES (Blood) |
| P62 | CH | | M | 6 | | NO | NO | NO | | NO | NO | NO | | | 12 | | L5/R3 | ELISA (Serum), WES (Blood) |
| P63 | CH | | M | 6 | | NO | NO | NO | | NO | NO | NO | | | 6 | | L4/R3 | WES (Blood) |
| P64 | CH | | M | 8 | | NO | NO | NO | | NO | NO | NO | | | 24 | | L4/R3 | ELISA (Serum), WES (Blood) |
| P65 | CH | | M | 8 | | NO | NO | NO | | NO | NO | NO | | | 12 | | L5/R3 | LISA (Serum), WES (Blood) |
| P66 | CH | | M | 9 | | NO | NO | NO | | NO | NO | NO | | | 60 | | L3/R3 | WES (Blood) |
| P67 | CH | | M | 9 | | NO | NO | NO | | NO | NO | NO | | | 10 | | L4/R4 | ELISA (Serum), WES (Blood) |
| P68 | CH | | M | 11 | | NO | NO | NO | | NO | NO | NO | | | 10 | | L2/R2 | cell assays (Serum), WES (Blood) |
| P69 | CH | | M | 11 | | NO | NO | NO | | NO | NO | NO | | | 12 | | L4/R5 | ell assays (Serum), WES (Blood) |
| P70 | CH | | M | 15 | | NO | NO | NO | | NO | NO | NO | | | 11 | | L2/R1 | cell assays (Serum), WES (Blood) |
| P71 | CH | | F | 6 | | NO | NO | NO | | NO | NO | NO | | | 1 | | L2/R3 | ELISA (Serum), WES (Blood) |
| P72 | CH | | F | 7 | | NO | NO | NO | | NO | NO | NO | | | 12 | | L3/R3 | WES (Blood) |
| P73 | CH | | F | 7 | | NO | NO | NO | | NO | NO | NO | | | 12 | | L5/R5 | WES (Blood) |
| P74 | CH | | F | 9 | | NO | NO | NO | | NO | NO | NO | | | 7 | | L3/R3 | ELISA(Serum) |
| P75 | CH | | F | 9 | | NO | NO | NO | | NO | NO | NO | | | 8 | | L3/R4 | ELISA (Serum), WES (Blood) |
| P76 | CH | | F | 9 | | NO | NO | NO | | NO | NO | NO | | | 6 | | L5/R3 | ELISA (Serum), WES (Blood) |
| P77 | CH | | F | 10 | | NO | NO | NO | | NO | NO | NO | | | 3 | | L3/R4 | ELISA (Serum), WES (Blood) |
| P78 | CH | | F | 10 | | NO | NO | NO | | NO | NO | NO | | | 6 | | L4/R4 | ELISA (Serum), WES (Blood) |
| P79 | CH | | F | 11 | | NO | NO | NO | | NO | NO | NO | | | 8 | | L5/R4 | ELISA (Serum), WES (Blood) |
| P80 | CH | | F | 11 | | NO | NO | NO | | NO | NO | NO | | | 6 | | L1/R3 | ELISA (Serum) |
| P81 | HEM | | M | 18 | | NO | NO | NO | | NO | NO | NO | | | 8 | | L3/R3 | untargeted metabolomics (Serum), WES (Blood) |
| P82 | HEM | | M | 23 | | NO | NO | NO | | NO | NO | NO | | | 6 | | L5/R3 | untargeted metabolomics (Serum) |
| P83 | HEM | | M | 27 | | NO | NO | NO | | NO | NO | NO | | | 6 | | L4/R3 | untargeted metabolomics, ELISA, cell assays (Serum), WES (Blood) |
| P84 | HEM | | M | 28 | | NO | NO | NO | | NO | 2 | NO | | | 9 | | L3/R2 | untargeted metabolomics, ELISA (Serum), WES (Blood) |
| P85 | HEM | | M | 32 | | NO | NO | NO | | NO | NO | NO | | | 8 | | L3/R3 | untargeted metabolomics (Serum), WES (Blood) |
| P86 | HEM | | M | 32 | | NO | NO | NO | | NO | NO | NO | | | 14 | | L3/R1 | untargeted metabolomics, ELISA, cell assays (Serum), WES (Blood) |
| P87 | HEM | | M | 44 | | NO | NO | NO | | NO | NO | NO | | | 9 | | L3/R1 | untargeted metabolomics, ELISA, cell assays (Serum), WES (Blood) |
| P88 | HEM | | M | 52 | | NO | NO | NO | | NO | 1 | 1 | | | 3 | | L3/R3 | untargeted metabolomics, ELISA (Serum) |
| P89 | HEM | | M | 40 | | NO | NO | NO | | NO | NO | NO | | | 6 | | L2/R3 | untargeted metabolomics (Serum), WES (Blood) |
| P90 | HEM | | M | 40 | | NO | NO | NO | | NO | 2 | 2 | | | 24 | | L5/R1 | untargeted metabolomics (Serum), WES (Blood) |
| P91 | HEM | | F | 18 | | NO | NO | NO | | NO | NO | NO | | | 2 | | L1/R1 | untargeted metabolomics, ELISA (Serum), WES (Blood) |
| P92 | HEM | | F | 19 | | NO | NO | NO | | NO | NO | NO | | | 12 | | L3/R3 | untargeted metabolomics (Serum), WES (Blood) |
| P93 | HEM | | F | 26 | | NO | NO | NO | | NO | NO | NO | | | 5 | | L1/R2 | untargeted metabolomics (Serum), WES (Blood) |
| P94 | HEM | | F | 34 | | NO | NO | NO | | NO | NO | NO | | | 60 | | L3/R5 | untargeted metabolomics (Serum), WES (Blood) |
| P95 | HEM | | F | 35 | | NO | NO | NO | | NO | NO | NO | | | 24 | | L1/R1 | untargeted metabolomics, ELISA (Serum), WES (Blood) |
| P96 | HEM | | F | 38 | | NO | NO | NO | | NO | NO | NO | | | 96 | | L3/R2 | untargeted metabolomics, ELISA (Serum), WES (Blood) |
| P97 | HEM | | F | 40 | | NO | NO | NO | | NO | NO | NO | | | 2 | | L1/R3 | untargeted metabolomics (Serum) |
| P98 | HEM | | F | 42 | | NO | NO | NO | | NO | NO | NO | | | 10 | | L3/R3 | untargeted metabolomics (Serum), WES (Blood) |
| P99 | HEM | | F | 43 | | NO | NO | NO | | NO | NO | NO | | | 5 | | L3/R2 | untargeted metabolomics, ELISA (Serum), WES (Blood) |
| P100 | IS | | M | 18 | | NO | NO | NO | | NO | NO | NO | | | 24 | | L3/R3 | untargeted metabolomics, ELISA, cell assays (Serum), WES (Blood) |
| P101 | IS | | M | 20 | | NO | NO | NO | | NO | NO | NO | | | 12 | | L3/R3 | untargeted metabolomics, cell assays (Serum), WES (Blood) |
| P102 | IS | | M | 31 | | NO | NO | NO | | NO | NO | 1 | | | 12 | | L3/R3 | untargeted metabolomics (Serum), WES (Blood) |
| P103 | IS | | M | 34 | | NO | NO | NO | | NO | 2 | NO | | | 3 | | L1/R2 | untargeted metabolomics, ELISA (Serum), WES (Blood) |
| P104 | IS | | M | 35 | | NO | NO | NO | | NO | 1 | NO | | | 24 | | L1/R2 | untargeted metabolomics, ELISA (Serum) |
| P105 | IS | | M | 35 | | NO | NO | NO | | NO | NO | NO | | | 2 | | L2/R3 | untargeted metabolomics, ELISA (Serum), WES (Blood) |
| P106 | IS | | M | 38 | | NO | NO | NO | | NO | 1 | NO | | | 9 | | L3/R5 | untargeted metabolomics (Serum), WES (Blood) |
| P107 | IS | | M | 38 | | NO | NO | NO | | NO | NO | NO | | | 3 | | L3/R3 | untargeted metabolomics (Serum), WES (Blood) |
| P108 | IS | | M | 38 | | NO | NO | NO | | NO | NO | NO | | | 12 | | L1/R3 | untargeted metabolomics, ELISA, cell assays (Serum) |
| P109 | IS | | M | 42 | | NO | NO | NO | | NO | NO | NO | | | 4 | | L2/R2 | untargeted metabolomics (Serum), WES (Blood) |
| P110 | IS | | F | 19 | | NO | NO | NO | | NO | NO | NO | | | 6 | | L3/R5 | untargeted metabolomics (Serum), WES (Blood) |
| P111 | IS | | F | 31 | | NO | NO | NO | | NO | NO | NO | | | 3 | | L3/R1 | untargeted metabolomics, ELISA (Serum), WES (Blood) |
| P112 | IS | | F | 32 | | NO | NO | NO | | NO | NO | NO | | | 4 | | L3/R3 | untargeted metabolomics (Serum), WES (Blood) |
| P113 | IS | | F | 34 | | NO | NO | NO | | NO | NO | NO | | | 4 | | L3/R2 | untargeted metabolomics (Serum), WES (Blood) |
| P114 | IS | | F | 34 | | NO | NO | NO | | NO | NO | NO | | | 4 | | L3/R2 | untargeted metabolomics, ELISA (Serum), WES (Blood) |
| P115 | IS | | F | 35 | | NO | NO | NO | | NO | NO | NO | | | 3 | | L2/R3 | untargeted metabolomics (Serum), WES (Blood) |
| P116 | IS | | F | 38 | | NO | NO | NO | | NO | NO | NO | | | 5 | | L5/R3 | untargeted metabolomics, ELISA (Serum), WES (Blood) |
| P117 | IS | | F | 39 | | NO | NO | NO | | NO | NO | NO | | | 8 | | L4/R3 | untargeted metabolomics, ELISA (Serum), WES (Blood) |
| P118 | IS | | F | 43 | | NO | NO | NO | | NO | NO | NO | | | 5 | | L3/R4 | untargeted metabolomics (Serum), WES (Blood) |
| P119 | IS | | F | 30 | | NO | NO | NO | | NO | 2 | NO | | | 7 | | L2/R3 | untargeted metabolomics, ELISA (Serum), WES (Blood) |
| P120 | CH | | F | 7 | | NO | NO | NO | | NO | NO | NO | | | 6 | | L3/R4 | ELISA (Serum) |
| P121 | CH | | F | 9 | | NO | NO | NO | | NO | NO | NO | | | 4 | | L2/R2 | ELISA (Serum) |
| P122 | CH | | F | 10 | | NO | NO | NO | | NO | NO | NO | | | 5 | | L4/R5 | ELISA (Serum) |
| P123 | CH | | F | 6 | | NO | NO | NO | | NO | NO | NO | | | 6 | | L2/R1 | ELISA (Serum) |
| P124 | CH | | M | 11 | | NO | NO | NO | | NO | NO | NO | | | 12 | | L2/R3 | ELISA (Serum) |
| P125 | CH | | F | 12 | | NO | NO | NO | | NO | NO | NO | | | 8 | | L1/R4 | ELISA (Serum) |
| P126 | CH | | F | 15 | | NO | NO | NO | | NO | NO | NO | | | 3 | | L2/R2 | ELISA (Serum) |
| P127 | CH | | F | 16 | | NO | NO | NO | | NO | NO | NO | | | 7 | | L4/R3 | ELISA (Serum) |
| P128 | IS | | M | 50 | | NO | NO | NO | | NO | 1 | NO | | | 12 | | L3/R2 | ELISA (Serum) |
| P129 | IS | | M | 35 | | NO | NO | NO | | NO | NO | NO | | | 6 | | L5/R4 | ELISA (Serum) |
| P130 | IS | | M | 37 | | NO | NO | NO | | NO | 2 | 1 | | | 8 | | L3/R6 | ELISA (Serum) |
| P131 | IS | | M | 43 | | NO | NO | NO | | NO | NO | NO | | | 12 | | L3/R2 | ELISA (Serum) |
| P132 | IS | | F | 43 | | NO | NO | NO | | NO | NO | NO | | | 7 | | L3/R5 | ELISA (Serum) |
| P133 | IS | | F | 48 | | NO | NO | NO | | NO | NO | NO | | | 9 | | L2/R4 | ELISA (Serum) |
| P134 | IS | | M | 46 | | NO | NO | NO | | NO | 1 | NO | | | 10 | | L1/R2 | ELISA (Serum) |
| P135 | IS | | M | 46 | | NO | NO | NO | | NO | 2 | NO | | | 15 | | L5/R3 | ELISA (Serum) |
| P136 | HEM | | F | 32 | | NO | NO | NO | | NO | NO | NO | | | 6 | | L5/R3 | ELISA (Serum) |
| P137 | HEM | | F | 33 | | NO | NO | NO | | NO | NO | NO | | | 12 | | L2/R3 | ELISA (Serum) |
| P138 | HEM | | F | 53 | | NO | NO | NO | | NO | NO | NO | | | 9 | | L2/R3 | ELISA (Serum) |
| P139 | HEM | | F | 36 | | NO | NO | NO | | NO | NO | NO | | | 4 | | L4/R3 | ELISA (Serum) |
| P140 | HEM | | M | 34 | | NO | NO | NO | | NO | NO | NO | | | 8 | | L3/R1 | ELISA (Serum) |
| P141 | HEM | | F | 50 | | NO | NO | NO | | NO | NO | NO | | | 3 | | L3/R5 | ELISA (Serum) |
| P142 | HEM | | F | 54 | | NO | NO | NO | | NO | NO | NO | | | 6 | | L3/R3 | ELISA (Serum) |
| P143 | HEM | | M | 55 | | NO | NO | NO | | NO | 1 | 5 | | | 12 | | L1/R2 | ELISA (Serum) |
| P144 | HEM | | F | 55 | | NO | NO | NO | | NO | NO | NO | | | 8 | | L3/R5 | ELISA (Serum) |
| P145 | IS | | M | 46 | | NO | NO | NO | | NO | NO | NO | | | 2 | | L2/R3 | RNA seq (MCA) |
| P146 | IS | | F | 32 | | NO | NO | NO | | NO | 1 | NO | | | 1 | | L3/R5 | RNA seq (STA) |
| P147 | IS | | M | 51 | | NO | NO | NO | | NO | 1 | 1 | | | 3 | | L3/R3 | RNA seq (MCA) |
| P148 | IS | | M | 47 | | NO | NO | NO | | NO | 1 | | | 1 | 12 | | L1/R3 | RNA seq (MCA, STA) |
| P149 | IS | | M | 30 | | NO | NO | NO | | NO | 1 | NO | | | 4 | | L2/R2 | RNA seq (MCA) |
| P150 | HEM | | F | 34 | | NO | NO | NO | | NO | NO | NO | | | 6 | | L3/R5 | RNA seq (MCA) |
| P151 | IS | | M | 46 | | NO | NO | NO | | NO | NO | NO | | | 3 | | L3/R1 | RNA seq (MCA, STA) |
| P152 | IS | | F | 35 | | NO | NO | NO | | NO | NO | NO | | | 4 | | L3/R3 | RNA seq (MCA) |
| P153 | IS | | F | 38 | | NO | NO | NO | | NO | NO | NO | | | 6 | | L3/R3 | RNA seq (STA) |
| P154 | IS | | M | 52 | | NO | NO | NO | | NO | NO | NO | | | 3 | | L3/R1 | RNA seq (STA) |
| P155 | HEM | | F | 35 | | NO | NO | NO | | NO | NO | NO | | | 5 | | L2/R3 | RNA seq (STA) |
| P156 | IS | | F | 51 | | NO | NO | NO | | NO | NO | NO | | | 36 | | L2/R3 | RNA seq (STA) |
| P157 | IS | | M | 43 | | NO | NO | NO | | NO | 1 | NO | | | 4 | | L3/R2 | RNA seq (MCA, STA) |
| P158 | IS | | F | 39 | | NO | NO | NO | | NO | NO | NO | | | 3 | | L2/R3 | RNA seq (MCA) |
| P159 | HEM | | M | 51 | | NO | NO | NO | | NO | NO | NO | | | 228 | | L3/R1 | RNA seq (STA) |
| P160 | AS | | F | 54 | | 10 | NO | NO | | NO | NO | NO | | | 6 | |  | untargeted metabolomics (Serum) |
| P161 | AS | M | | 47 | | 6 | NO | NO | 6 | | 20 | 20 | | | 4 | |  | untargeted metabolomics (Serum) |
| P162 | AS | F | | 56 | | 5 | NO | NO | 5 | | 30 | 30 | | | 12 | |  | untargeted metabolomics (Serum) |
| P163 | AS | M | | 47 | | 8 | NO | NO | 5 | | 15 | 15 | | | 3 | |  | untargeted metabolomics (Serum) |
| P164 | AS | M | | 57 | | NO | 8 | NO | NO | | NO | NO | | | 12 | |  | untargeted metabolomics (Serum) |
| P165 | AS | M | | 70 | | 25 | NO | NO | NO | | 40 | 40 | | | 6 | |  | untargeted metabolomics (Serum) |
| P166 | AS | F | | 55 | | 10 | NO | NO | NO | | NO | NO | | | 3 | |  | untargeted metabolomics (Serum) |
| P167 | AS | M | | 73 | | 20 | NO | 20 | 20 | | NO | 10 | | | 9 | |  | untargeted metabolomics (Serum) |
| P168 | AS | M | | 64 | | 10 | NO | NO | NO | | NO | 20 | | | 15 | |  | untargeted metabolomics (Serum) |
| P169 | AS | M | | 66 | | 15 | NO | NO | 15 | | 35 | 35 | | | 5 | |  | untargeted metabolomics (Serum) |
| P170 | AS | M | | 70 | | 20 | NO | NO | NO | | NO | NO | | | 6 | |  | untargeted metabolomics (Serum) |
| P171 | AS | F | | 69 | | 20 | NO | NO | NO | | NO | NO | | | 8 | |  | untargeted metabolomics (Serum) |
| P172 | AS | F | | 71 | | NO | 25 | NO | NO | | NO | NO | | | 10 | |  | untargeted metabolomics (Serum) |
| P173 | AS | F | | 59 | | 15 | NO | NO | 15 | | NO | NO | | | 6 | |  | untargeted metabolomics (Serum) |
| P174 | AS | F | | 61 | | NO | NO | NO | NO | | NO | 20 | | | 12 | |  | untargeted metabolomics (Serum) |
| P175 | AS | M | | 53 | | 2 | NO | NO | NO | | 25 | 25 | | | 7 | |  | untargeted metabolomics (Serum) |
| P176 | AS | M | | 51 | | 1 | NO | NO | 1 | | 20 | 20 | | | 4 | |  | untargeted metabolomics (Serum) |
| P177 | AS | F | | 64 | | 16 | NO | NO | NO | | NO | NO | | | 3 | |  | untargeted metabolomics (Serum) |
| P178 | AS | M | | 46 | | 15 | NO | NO | NO | | 20 | 20 | | | 6 | |  | untargeted metabolomics (Serum) |
| P179 | AS | M | | 50 | | 20 | NO | NO | NO | | 25 | 25 | | | 8 | |  | untargeted metabolomics (Serum) |
| P180 | TBI | M | | 30 | | 1 | NO | NO | NO | | NO | NO | | | 1 | |  | RNA seq (STA) |
| P181 | IPA | M | | 50 | | 1 | NO | NO | NO | | NO | NO | | | 2 | |  | RNA seq (STA) |
| P182 | AC | M | | 20 | | NO | NO | NO | NO | | NO | NO | | | 6 | |  | RNA seq (STA) |

MMD, moyamoya disease. CH, child; HEM, hemorrhage; IS, ischemia. AS, atherosclerotic stenosis. M, male; F, female. L, left; R, right. MCA, middle cerebral artery; STA, superficial temporal artery. ELISA, enzyme-linked immunosorbent assays; RNA seq, RNA sequencing. Duration of symptoms indicates the duration from the first symptom until the hospitalization. TBI, traumatic brain injury. IPA, intracranial pseudoaneurysm. AC, arachnoid cyst.

**Table S2. Demographic and clinical characteristics of the participants**

| Variables | Ischemic (n = 70) | Hemorrhagic (n = 61) | | | Children (n = 28) | | Atherosclerotic stenosis (n=20) | | Controls (n = 30) |  |
| --- | --- | --- | --- | --- | --- | --- | --- | --- | --- | --- |
| Sex (M:F) | 31:39 | 21:40 | | | 11:17 | | 12:8 | | 15:15 |  |
| Age (years) | 37.86 ± 9.57 | 36.62 ± 9.61 | | | 9.36 ± 2.88 | | 59.15±8.82 | | 35.43 ± 10.36 |  |
| Smoking history | 14 (20.0%) | 7 (11.5%) | | | 0 | | 9 (45%) | | 0 |  |
| Alcohol taking | 4 (5.7%) | 4 (6.6%) | | | 0 | | 12 (60%) | | 0 |  |
| Mean duration of symptoms ± standard deviation (months)  Suzuki Stage | 8.51 ± 7.41 | | 15.20 ± 32.04 | 15.20 ± 32.04 | | 7.25±3.46 | |  |  |  |
| Left |  |  | | |  | |  | |  |  |
| 1 | 9 (12.8%) | 9 (14.8%) | | | 2 (7.1%) | |  | |  |  |
| 2 | 12 (17.1%) | 15 (24.6%) | | | 7 (25.0%) | |  | |  |  |
| 3 | 37 (52.8%) | 24 (39.3%) | | | 7 (25.0%) | |  | |  |  |
| 4 | 6 (8.6%) | 5 (8.2%) | | | 7 (25.0%) | |  | |  |  |
| 5 | 6 (8.6%) | 8 (13.1%) | | | 5 (17.8%) | |  | |  |  |
| 6 | 0 | 0 | | | 0 | |  | |  |  |
| Right |  |  | | |  | |  | |  |  |
| 1 | 9 (12.8%) | 10 (16.4%) | | | 2 (7.1%) | |  | |  |  |
| 2 | 17 (24.3%) | 12 (19.7%) | | | 3 (10.7%) | |  | |  |  |
| 3 | 31 (44.3%) | 29 (47.5%) | | | 12 (42.8%) | |  | |  |  |
| 4 | 5 (7.1%) | 4 (6.6%) | | | 8 (28.6%) | |  | |  |  |
| 5 | 7 (10.0%) | 5 (8.2%) | | | 3 (10.7%) | |  | |  |  |
| 6 | 1 (1.4%) | 1 (1.6%) | | | 0 | |  | |  |  |

The demographic and clinical information of five groups of participants of different types of MMD and atherosclerotic stenosis.

**Table S****3**: **Patient information in untargeted metabolomics**

| Variables | Ischemic (n=20) | Hemorrhagic (n=20) | Atherosclerotic stenosis (n=20) | Controls (n=20) | Statistic P value |  |
| --- | --- | --- | --- | --- | --- | --- |
| Sex (M : F) | 10:10 | 10:10 | 12:8 | 10:10 | 0.958 |  |
| Age (years) | 33.05±7.39 | 33.65±9.65 | 59.15±8.82 | 32.00±4.13 | 0.000 |  |
| Smoking history | 4 (20%) | 3 (15%) | 9 (45%) | 0 | 0.000 |  |
| Alcohol taking | 1 (5%) | 2 (10%) | 12 (60%) | 0 | 0.000 |  |
| Mean duration of symptom±SD (months)  Suzuki Stage | 7.70±6.42 | 15.78±22.92 | 7.25±3.46 |  | 0.369 |  |
| Left |  |  |  |  |  |  |
| 1 | 3 (15%) | 4 (20%) |  |  |  |  |
| 2 | 4 (20%) | 2 (10%) |  |  |  |  |
| 3 | 11 (55%) | 11 (55%) |  |  |  |  |
| 4 | 1 (5%) | 1 (5%) |  |  |  |  |
| 5 | 1 (5%) | 2 (30%) |  |  |  |  |
| 6 | 0 | 0 |  |  |  |  |
| Right |  |  |  |  |  |  |
| 1 | 1 (5%) | 6 (30%) |  |  |  |  |
| 2 | 5 (25%) | 4 (20%) |  |  |  |  |
| 3 | 11 (55%) | 9 (45%) |  |  |  |  |
| 4 | 1 (5%) | 0 |  |  |  |  |
| 5 | 2 (10%) | 1 (5%) |  |  |  |  |
| 6 | 0 | 0 |  |  |  |  |

**Table S4: Patient information in targeted metabolomics**

| Variables | Ischemic (n=30) | Hemorrhagic (n= 29) | Controls (n=30) | Statistic P value |  |
| --- | --- | --- | --- | --- | --- |
| Sex (M : F) | 8:22 | 9:20 | 15:15 | 0.135 |  |
| Age (years) | 37.63±11.16 | 36.21±8.26 | 35.43±10.36 | 0.699 |  |
| Smoking history | 1 (3.3%) | 3 (10.3%) | 0 | 0.122 |  |
| Alcohol taking | 0 | 1 (3.4%) | 0 | 0.326 |  |
| Mean duration of symptoms±SD (months)  Suzuki Stage | 9.40±7.97 | 10.59±13.23 |  | 0.6774 |  |
| Left |  |  |  |  |  |
| 1 | 4 (13.3%) | 4 (13.8%) |  |  |  |
| 2 | 3 (10%) | 10 (34.5%) |  |  |  |
| 3 | 15 (50%) | 7 ( 24.1%) |  |  |  |
| 4 | 5 (16.7%) | 3 (10.3%) |  |  |  |
| 5 | 3 (10%) | 5 (17.2%) |  |  |  |
| 6 | 0 | 0 |  |  |  |
| Right |  |  |  |  |  |
| 1 | 6 (20%) | 2 (6.9%) |  |  |  |
| 2 | 7 (23.3%) | 7 (24.1%) |  |  |  |
| 3 | 12 (40%) | 14 (48.3%) |  |  |  |
| 4 | 2 (6.7%) | 4 (13.8%) |  |  |  |
| 5 | 3 (10%) | 1 (3.4%) |  |  |  |
| 6 | 0 | 1 (3.4%) |  |  |  |

**Table S5: Patient information in ELISA**

| Variables | Ischemic (n=20) | Hemorrhagic (n=20) | Children (n=20) | Controls (n=20) | Statistic P value |  |
| --- | --- | --- | --- | --- | --- | --- |
| Sex (M : F) | 12:8 | 7:13 | 5:15 | 10:10 | 0.115 |  |
| Age (years) | 38.80±7.95 | 40.55±10.85 | 9.60±2.66 | 32.00±4.13 | 0.000 |  |
| Smoking history | 7 (35%) | 3 (15%) | 0 | 0 | 0.001 |  |
| Alcohol taking | 1 (5%) | 2 (10%) | 0 | 0 | 0.611 |  |
| Mean duration of symptoms±SD (months)  Suzuki Stage | 10.20±6.85 | 12.10±20.40 | 7.70±4.88 |  | 0.464 |  |
| Left |  |  |  |  |  |  |
| 1 | 4 (20%) | 3 (15%) | 2 (10%) |  |  |  |
| 2 | 4 (20%) | 4 (20%) | 5 (25%) |  |  |  |
| 3 | 8 (40%) | 10 (50%) | 4 (20%) |  |  |  |
| 4 | 1 (5%) | 2 (10%) | 5 (25%) |  |  |  |
| 5 | 3 (15%) | 1 (5%) | 4 (20%) |  |  |  |
| 6 | 0 | 0 | 0 |  |  |  |
| Right |  |  |  |  |  |  |
| 1 | 1 (5%) | 6 (30%) | 1 (5%) |  |  |  |
| 2 | 8 (40%) | 4 (20%) | 2 (10%) |  |  |  |
| 3 | 7 (35%) | 8 (40%) | 9 (45%) |  |  |  |
| 4 | 2 (10%) | 0 | 7 (35%) |  |  |  |
| 5 | 1 (5%) | 2 (10%) | 1 (5%) |  |  |  |
| 6 | 1 (5%) | 0 | 0 |  |  |  |

**Table S6: Patient information in cell assays**

| Variables | Ischemic (n=3) | | Hemorrhagic (n=3) | Children (n=3) | Controls (n=3) | Statistic P value |  |
| --- | --- | --- | --- | --- | --- | --- | --- |
| Sex (M : F) | 3:0 | | 3:0 | 3:0 | 3:0 | 1 |  |
| Age (years) | 25.33±11.02 | | 34.67±8.33 | 12.33±2.31 | 27.67±1.15 | 0.052 |  |
| Smoking history | 0 | | 0 | 0 | 0 | 1 |  |
| Alcohol taking | 0 | | 0 | 0 | 0 | 1 |  |
| Mean duration of symptoms±SD (months) | 16.00±6.93 | | 9.67±4.04 | 11.00±1.00 |  | 0.276 |  |
| LPC 16:1-2  relative level | |  |  |  |  |  |  |
| 1 | | 0.123996 | 15.53938 | 25.51889 | 57.70173 |  |  |
| 2 | | 0.214561 | 16.10639 | 28.39658 | 30.09621 |  |  |
| 3 | | 0.204033 | 22.00622 | 14.41109 | 50.52026 |  |  |
| LPC 22:6  relative level | |  |  |  |  |  |  |
| 1 | | 323597 | 302912.4 | 453732.7 | 800701.2 |  |  |
| 2 | | 230033.6 | 292718.7 | 562929.2 | 658060.5 |  |  |
| 3 | | 382518.6 | 249117.2 | 461913.6 | 734608.2 |  |  |

**Table S7: The top 20 differential metabolites (positive ion) for is ischemic moyamoya disease (IS) and healthy controls (HC)**

| No. | VarID | Compounds | FC | log2(FC) | raw.pvalue | -log10(p) |
| --- | --- | --- | --- | --- | --- | --- |
| 1 | V501 | LPC 16:1-2 | 0.051344 | -4.2837 | 3.75E-08 | 7.4258 |
| 2 | V774 | PE 38:5e | 0.65533 | -0.6097 | 1.17E-06 | 5.9304 |
| 3 | V49 | 1H-Indole-3-Acetic Acid | 0.43636 | -1.1964 | 2.75E-06 | 5.5613 |
| 4 | V693 | PC 18:0_20:4 | 0.6244 | -0.67946 | 0.00053508 | 3.2716 |
| 5 | V701 | PC 19:0_20:4 | 1.2484 | 0.32007 | 0.0016416 | 2.7847 |
| 6 | V225 | Asp-phe | 1.7923 | 0.84183 | 0.0024949 | 2.603 |
| 7 | V472 | L-erythro-4-Hydroxyglutamate | 1.5832 | 0.66282 | 0.0033773 | 2.4714 |
| 8 | V443 | Indoline | 0.19437 | -2.3632 | 0.0045755 | 2.3396 |
| 9 | V478 | linolenic acid | 0.51632 | -0.95366 | 0.0050407 | 2.2975 |
| 10 | V10 | (+/-)-Equol | 0.76621 | -0.3842 | 0.0051122 | 2.2914 |
| 11 | V275 | cis-4,7,10,13,16-docosapentaenoic acid | 0.54479 | -0.87621 | 0.0052069 | 2.2834 |
| 12 | V613 | Mono-(2-ethyl-5-hydroxyhexyl) phthalate | 0.69543 | -0.52403 | 0.0063507 | 2.1972 |
| 13 | V44 | 17beta-Nio-5a-androstane | 0.80769 | -0.30812 | 0.0094999 | 2.0223 |
| 14 | V531 | LPC 3:1 | 0.72781 | -0.45836 | 0.0095685 | 2.0192 |
| 15 | V7 | L-Phenylalanine-d5 | 0.83669 | -0.25723 | 0.010264 | 1.9887 |
| 16 | V30 | 1-(4-Hydroxyphenyl)-1-decene-3,5-dione | 0.57446 | -0.79973 | 0.011991 | 1.9212 |
| 17 | V841 | sn-Glycero-3-phosphocholine | 0.75786 | -0.4 | 0.01246 | 1.9045 |
| 18 | V470 | L-Dopa | 0.80791 | -0.30773 | 0.013561 | 1.8677 |
| 19 | V731 | PC O-32:1-sn2 | 0.54664 | -0.87134 | 0.014409 | 1.8414 |
| 20 | V237 | beta-Linoleic acid | 0.69807 | -0.51857 | 0.015408 | 1.8122 |

**Table S8: The top 20 differential metabolites (negative ion) for is ischemic moyamoya disease (IS) and healthy controls (HC)**

| No. | VarID | Compounds | FC | log2(FC) | raw.pvalue | -log10(p) |
| --- | --- | --- | --- | --- | --- | --- |
| 1 | V102 | S-Sulfo-L-cysteine | 0.46469 | -1.1057 | 1.56E-08 | 7.808 |
| 2 | V416 | LPC 18:2 | 0.63373 | -0.65806 | 2.55E-07 | 6.5938 |
| 3 | V118 | L-Cysteinesulfinic acid | 0.26964 | -1.8909 | 3.36E-07 | 6.4735 |
| 4 | V72 | Lactic acid | 1.6322 | 0.70685 | 3.66E-07 | 6.4361 |
| 5 | V73 | Lactic acid 1 | 1.6034 | 0.68114 | 9.96E-07 | 6.0019 |
| 6 | V74 | Lactic acid 2 | 1.5706 | 0.65135 | 6.02E-06 | 5.2201 |
| 7 | V449 | 8(R)-Hydroxy-(5Z,9E,11Z,14Z)-eicosatetraenoic acid | 8.9144 | 3.1561 | 1.46E-05 | 4.8352 |
| 8 | V101 | sn-Glycero-3-phosphocholine | 0.60284 | -0.73015 | 1.77E-05 | 4.7519 |
| 9 | V317 | Biliverdin | 6.1676 | 2.6247 | 2.54E-05 | 4.5958 |
| 10 | V452 | FA 5:20 | 3.9604 | 1.9857 | 3.73E-05 | 4.4282 |
| 11 | V285 | (R)-2-hydroxystearic acid | 0.38381 | -1.3815 | 4.04E-05 | 4.3931 |
| 12 | V418 | LPE 20:2 | 0.6925 | -0.53012 | 7.67E-05 | 4.1152 |
| 13 | V220 | Azelaic acid | 0.70968 | -0.49477 | 0.000212 | 3.6727 |
| 14 | V128 | 4-Hydroxybutanoic acid | 2.2709 | 1.1832 | 0.000229 | 3.6406 |
| 15 | V404 | LPE 18:2 | 0.50612 | -0.98244 | 0.000242 | 3.6167 |
| 16 | V185 | Indoxyl sulfate | 0.4567 | -1.1307 | 0.000328 | 3.4841 |
| 17 | V171 | Asp-phe | 1.9471 | 0.9613 | 0.0004 | 3.398 |
| 18 | V396 | 1-Hydroxy-2-(9Z,12Z-octadecadienoyl)-sn-glycero-3-phosphoethanolamine | 0.53123 | -0.9126 | 0.000435 | 3.3611 |
| 19 | V442 | 9,10-DHOME | 0.4495 | -1.1536 | 0.000533 | 3.2736 |
| 20 | V222 | (-)-Usnic acid | 0.48309 | -1.0496 | 0.000629 | 3.2012 |

**Table S9: The top 10 differential metabolites (positive ion) for hemorrhagic moyamoya disease (HEM) and healthy controls (HC)**

| No. | varID | Compounds | FC | log2(FC) | raw.pval | -log10(p) |
| --- | --- | --- | --- | --- | --- | --- |
|  | V774 | PE 38:5e | 0.61161 | -0.70932 | 7.78E-08 | 7.1093 |
| 2 | V225 | Asp-phe | 1.7736 | 0.82668 | 0.000624 | 3.205 |
| 3 | V634 | N-Acetylmethionine | 0.77096 | -0.37527 | 0.003575 | 2.4468 |
| 4 | V7 | L-Phenylalanine-d5 | 0.84365 | -0.24528 | 0.004553 | 2.3417 |
| 5 | V11 | (+/-)-Octopamine | 0.6057 | -0.72333 | 0.005152 | 2.288 |
| 6 | V30 | 1-(4-Hydroxyphenyl)-1-decene-3,5-dione | 0.54646 | -0.87182 | 0.00716 | 2.1451 |
| 7 | V472 | L-erythro-4-Hydroxyglutamate | 1.371 | 0.45523 | 0.007886 | 2.1031 |
| 8 | V717 | PC 36:3 | 1.2355 | 0.30511 | 0.008351 | 2.0783 |
| 9 | V693 | PC 18:0_20:4 | 0.7794 | -0.35956 | 0.008677 | 2.0616 |
| 10 | V502 | LPC 17:0 | 0.69499 | -0.52494 | 0.009981 | 2.0008 |

**Table S10: The top 10 differential metabolites (negative ion) for hemorrhagic moyamoya disease (HEM) and healthy controls (HC)**

| No. | varID | Compounds | FC | log2(FC) | raw.pval | -log10(p) |
| --- | --- | --- | --- | --- | --- | --- |
| 1 | V416 | LPC 18:2 | 0.66171 | -0.59572 | 2.05E-06 | 5.6877 |
| 2 | V418 | LPE 20:2 | 0.65014 | -0.62118 | 5.65E-06 | 5.2476 |
| 3 | V452 | FA 5:20 | 4.0579 | 2.0207 | 1.15E-05 | 4.938 |
| 4 | V72 | Lactic acid | 1.456 | 0.54197 | 2.45E-05 | 4.6109 |
| 5 | V449 | 8(R)-Hydroxy-(5Z,9E,11Z,14Z)-eicosatetraenoic acid | 7.7674 | 2.9574 | 2.60E-05 | 4.5843 |
| 6 | V227 | Tetrahydroaldosterone-3-glucuronide | 0.51055 | -0.96988 | 2.97E-05 | 4.5267 |
| 7 | V73 | Lactic acid 1 | 1.4536 | 0.53967 | 4.48E-05 | 4.3492 |
| 8 | V422 | Sulfaphenazole | 0.54433 | -0.87744 | 6.42E-05 | 4.1922 |
| 9 | V74 | Lactic acid 2 | 1.4278 | 0.51382 | 0.000161 | 3.7938 |
| 10 | V69 | Inosine | 0.6914 | -0.53242 | 0.000171 | 3.7672 |

**Table S11: The top 10 differential metabolites (positive ion) for moyamoya disease (IS&HEM) and healthy controls (HC)**

| No. | varID | Compounds | FC | log2(FC) | raw.pval | -log10(p) |
| --- | --- | --- | --- | --- | --- | --- |
| 1 | V774 | PE 38:5e | 0.63294 | -0.65987 | 8.32E-09 | 8.0799 |
| 2 | V225 | Asp-phe | 1.7827 | 0.83409 | 4.24E-05 | 4.373 |
| 3 | V49 | 1H-Indole-3-Acetic Acid | 0.57912 | -0.78807 | 0.000165 | 3.7836 |
| 4 | V693 | PC 18:0_20:4 | 0.70379 | -0.50678 | 0.000174 | 3.7603 |
| 5 | V472 | L-erythro-4-Hydroxyglutamate | 1.4745 | 0.56023 | 0.000751 | 3.1244 |
| 6 | V701 | PC 19:0_20:4 | 1.2227 | 0.29009 | 0.000794 | 3.1004 |
| 7 | V167 | ACar 16:3 | 3.0187 | 1.5939 | 0.001407 | 2.8517 |
| 8 | V634 | N-Acetylmethionine | 0.78332 | -0.35233 | 0.002316 | 2.6353 |
| 9 | V7 | L-Phenylalanine-d5 | 0.84026 | -0.2511 | 0.003665 | 2.4359 |
| 10 | V501 | LPC 16:1-2 | 0.55129 | -0.85911 | 0.003784 | 2.4221 |

**Table S12: The top 10 differential metabolites (negative ion) for moyamoya disease (IS&HEM) and healthy controls (HC)**

| No. | varID | Compounds | FC | log2(FC) | raw.pval | -log10(p) |
| --- | --- | --- | --- | --- | --- | --- |
| 1 | V449 | 8(R)-Hydroxy-(5Z,9E,11Z,14Z)-eicosatetraenoic acid | 8.3269 | 3.0578 | 8.33E-10 | 9.0794 |
| 2 | V452 | FA 5:20 | 4.0103 | 2.0037 | 1.13E-09 | 8.9488 |
| 3 | V416 | LPC 18:2 | 0.64806 | -0.62579 | 9.69E-09 | 8.0139 |
| 4 | V72 | Lactic acid | 1.542 | 0.62476 | 4.91E-08 | 7.3088 |
| 5 | V73 | Lactic acid 1 | 1.5267 | 0.61041 | 1.12E-07 | 6.9498 |
| 6 | V102 | S-Sulfo-L-cysteine | 0.56922 | -0.81293 | 8.16E-07 | 6.0882 |
| 7 | V74 | Lactic acid 2 | 1.4975 | 0.58255 | 1.33E-06 | 5.8765 |
| 8 | V418 | LPE 20:2 | 0.6708 | -0.57604 | 1.68E-06 | 5.7744 |
| 9 | V317 | Biliverdin | 4.8062 | 2.2649 | 2.72E-06 | 5.5656 |
| 10 | V46 | D-Galactose/D-Glucose | 0.83795 | -0.25506 | 3.65E-05 | 4.4372 |

**Table S13: The top 10 differential metabolites (positive ion) for ischemic moyamoya disease (IS) and hemorrhagic moyamoya disease (HEM)**

| No. | varID | Compounds | FC | log2(FC) | raw.pval | -log10(p) |
| --- | --- | --- | --- | --- | --- | --- |
| 1 | V501 | LPC 16:1-2 | 0.049973 | -4.3227 | 2.47E-10 | 9.6069 |
| 2 | V49 | 1H-Indole-3-Acetic Acid | 0.61023 | -0.71258 | 0.000963 | 3.0163 |
| 3 | V613 | Mono-(2-ethyl-5-hydroxyhexyl) phthalate | 0.6508 | -0.61971 | 0.002502 | 2.6017 |
| 4 | V463 | L-Carnitine | 1.3794 | 0.464 | 0.004976 | 2.3031 |
| 5 | V10 | (+/-)-Equol | 0.7777 | -0.36272 | 0.006999 | 2.155 |
| 6 | V424 | Guanidinoacetate | 0.67217 | -0.57311 | 0.007797 | 2.1081 |
| 7 | V686 | PC 16:1_20:4 | 0.68329 | -0.54943 | 0.009195 | 2.0365 |
| 8 | V791 | Pirimicarb | 1.4062 | 0.49177 | 0.011404 | 1.9429 |
| 9 | V719 | PC 36:4-2 | 0.66475 | -0.58911 | 0.011801 | 1.9281 |
| 10 | V702 | PC 20:2 | 0.71698 | -0.48 | 0.013996 | 1.854 |

**Table S14: The top 10 differential metabolites (negative ion) for ischemic moyamoya disease (IS) and hemorrhagic moyamoya disease (HEM)**

| No. | varID | Compounds | FC | log2(FC) | raw.pval | -log10(p) |
| --- | --- | --- | --- | --- | --- | --- |
| 1 | V408 | LPC 22:6 | 1.3543 | 0.43754 | 0.002223 | 2.6531 |
| 2 | V102 | S-Sulfo-L-cysteine | 0.69483 | -0.52526 | 0.003832 | 2.4166 |
| 3 | V54 | D-Pantethine | 0.48877 | -1.0328 | 0.007063 | 2.151 |
| 4 | V69 | Inosine | 1.2349 | 0.30444 | 0.016178 | 1.7911 |
| 5 | V475 | FAHFA 26:0 | 1.6324 | 0.70699 | 0.016965 | 1.7705 |
| 6 | V214 | D-Erythrose 4-phosphate | 0.37423 | -1.418 | 0.019929 | 1.7005 |
| 7 | V171 | Asp-phe | 1.5006 | 0.58553 | 0.023691 | 1.6254 |
| 8 | V359 | LPA 20:4 | 0.52299 | -0.93515 | 0.032955 | 1.4821 |
| 9 | V317 | Biliverdin | 1.7573 | 0.81339 | 0.033716 | 1.4722 |
| 10 | V341 | Pangamic acid/simvastatin hydroxy acid | 0.71 | -0.49412 | 0.041593 | 1.381 |

**Table S15: The top 10 differential metabolites (positive ion) for AS (atherosclerotic stenosis) and healthy controls (HC)**

| No. | varID | Compounds | FC | log2(FC) | raw.pval | -log10(p) |
| --- | --- | --- | --- | --- | --- | --- |
| 1 | V251 | Gamma-Butyrolactone | 9.79E-05 | -13.318 | 6.35E-20 | 19.197 |
| 2 | V11 | (23E)-5,25-Dihydroxy-4,4-dimethylergosta-8,23-diene-3,22-dione | 145050 | 17.146 | 8.19E-20 | 19.087 |
| 3 | V276 | Hodgkinsine | 976770 | 19.898 | 1.12E-19 | 18.951 |
| 4 | V57 | 3-O-Acetylepisamarcandin | 8661800 | 23.046 | 7.56E-17 | 16.121 |
| 5 | V35 | 1-Oleoyl-Sn-Glycero-3-Phosphocholine | 15.003 | 3.9072 | 1.20E-15 | 14.919 |
| 6 | V473 | PC(O-16:2(9E,10E)/0:0)[U] | 631210 | 19.268 | 1.57E-14 | 13.805 |
| 7 | V532 | beta-Sinensal | 151430 | 17.208 | 2.56E-14 | 13.592 |
| 8 | V498 | Pro-Leu | 0.046376 | -4.4305 | 5.20E-14 | 13.284 |
| 9 | V340 | LPC 20:5 | 1285200 | 20.294 | 1.58E-13 | 12.801 |
| 10 | V327 | LPC 18:2-sn1 | 15.581 | 3.9617 | 3.62E-13 | 12.441 |

**Table S16: The top 10 differential metabolites (positive ion) for AS (atherosclerotic stenosis) and ischemic moyamoya disease (IS)**

| No. | varID | Compounds | FC | log2(FC) | raw.pval | -log10(p) |
| --- | --- | --- | --- | --- | --- | --- |
| 1 | V340 | LPC 20:5 | 9.6578 | 3.2717 | 4.51E-14 | 13.346 |
| 2 | V401 | Ne,Ne-Dimethyl-L-lysine | 8.9455 | 3.1612 | 1.50E-13 | 12.824 |
| 3 | V230 | Ecgonine | 12.758 | 3.6733 | 5.10E-13 | 12.292 |
| 4 | V551 | Uric acid | 11.213 | 3.4871 | 5.63E-13 | 12.25 |
| 5 | V331 | LPC 18:3-sn2 | 8.0195 | 3.0035 | 7.98E-13 | 12.098 |
| 6 | V469 | PC(20:5(5Z,8Z,11Z,14Z,17Z)/0:0) | 8.482 | 3.0844 | 1.00E-12 | 11.999 |
| 7 | V473 | PC(O-16:2(9E,10E)/0:0)[U] | 7.8946 | 2.9809 | 1.50E-12 | 11.823 |
| 8 | V530 | SM d34:1 | 8.6224 | 3.1081 | 2.87E-12 | 11.542 |
| 9 | V364 | LPE 19:2 | 9.3539 | 3.2256 | 3.43E-12 | 11.465 |
| 10 | V532 | beta-Sinensal | 8.0225 | 3.0041 | 3.90E-12 | 11.408 |

**Table S17: The top 10 differential metabolites (negative ion) for AS (atherosclerotic stenosis) and hemorrhagic moyamoya disease (HEM)**

| No. | varID | Compounds | FC | log2(FC) | raw.pval | -log10(p) |
| --- | --- | --- | --- | --- | --- | --- |
| 1 | V473 | PC(O-16:2(9E,10E)/0:0)[U] | 18.416 | 4.2029 | 6.44E-18 | 17.191 |
| 2 | V340 | LPC 20:5 | 14.943 | 3.9014 | 1.07E-15 | 14.973 |
| 3 | V532 | beta-Sinensal | 10.26 | 3.359 | 1.47E-15 | 14.834 |
| 4 | V276 | Hodgkinsine | 9.2169 | 3.2043 | 3.65E-15 | 14.438 |
| 5 | V35 | 1-Oleoyl-Sn-Glycero-3-Phosphocholine | 10.132 | 3.3408 | 6.63E-15 | 14.179 |
| 6 | V401 | Ne,Ne-Dimethyl-L-lysine | 11.959 | 3.58 | 1.29E-14 | 13.889 |
| 7 | V195 | Deoxycorticosterone | 14.32 | 3.84 | 1.80E-14 | 13.745 |
| 8 | V530 | SM d34:1 | 15.971 | 3.9974 | 5.15E-14 | 13.288 |
| 9 | V550 | UCM707 | 10.074 | 3.3325 | 8.46E-14 | 13.073 |
| 10 | V122 | Acetylepisamarcandin | 10.386 | 3.3766 | 1.05E-13 | 12.977 |

**Table S18: Statistical results of Targeted metabolomics**

| Name | IS | HEM | HC | pvalue(IS:HEM) | pvalue(IS:HC ) | pvalue(HEM:HC) |
| --- | --- | --- | --- | --- | --- | --- |
| LPC 16:0 | 493085100.8 | 581319604.8 | 575196194.9 | 0.347860394 | 0.397040633 | 0.956862684 |
| LPC 16:0 2 | 267922581.4 | 314766760.6 | 308988465.8 | 0.353275195 | 0.441573803 | 0.88477218 |
| LPE 16:0 | 383993.8817 | 460924.6028 | 368015.4815 | 0.434478345 | 0.747181807 | 0.280509096 |
| LPE 16:0 2 | 117813.6059 | 128655.7528 | 123632.8794 | 0.830670266 | 0.733267891 | 0.952625341 |
| LPC 14:0 | 4252963.424 | 5637836.647 | 5384520.93 | 0.149632121 | 0.287889996 | 0.668925311 |
| LPC 15:0 | 2604262.289 | 3692699.619 | 3469257.139 | 0.025568854 | 0.091075069 | 0.662320397 |
| LPC 16:1 | 11345412.04 | 14902670.83 | 14927288.87 | 0.147118753 | 0.138707592 | 0.983616585 |
| LPC 16:2 | 49547.58799 | 138678.0507 | 129863.7205 | 7.36567E-05 | 0.000420331 | 0.671214523 |
| LPC 18:0 | 188864010.7 | 221805203.4 | 224698936.3 | 0.405931597 | 0.372664007 | 0.873278971 |
| LPC 18:1 | 106573604.2 | 117391663.4 | 117285765.1 | 0.727587835 | 0.733766883 | 0.987352739 |
| LPC 18:2 | 275376955.8 | 275154715.2 | 276964931.9 | 0.886337772 | 0.851440077 | 0.941548965 |
| LPC 18:3 | 5040310.044 | 4899803.94 | 4912626.754 | 0.768369438 | 0.700072836 | 0.856664968 |
| LPC 18:4 | 3058.622613 | 8174.041624 | 32901.68553 | 0.325499353 | 0.000126145 | 0.001868809 |
| LPC 19:0 | 473641.559 | 577996.236 | 516231.3114 | 0.402766907 | 0.702695626 | 0.628161912 |
| LPC 20:0 | 619072.4753 | 850262.1284 | 963588.5275 | 0.192311273 | 0.056691295 | 0.326958204 |
| LPC 20:1 | 1173949.664 | 1370500.286 | 1465501.563 | 0.46966434 | 0.283997458 | 0.590016318 |
| LPC 20:2 | 2409730.18 | 2558837.607 | 2436386.002 | 0.898175043 | 0.900072372 | 0.745345022 |
| LPC 20:3 | 13926757.86 | 13604241.23 | 13881459.07 | 0.80843241 | 0.750763957 | 0.894854602 |
| LPC 20:4 | 56912105.86 | 56892637.75 | 55303390.05 | 0.87811612 | 0.752629105 | 0.810753784 |
| LPC 20:5 | 3111495.766 | 2857443.113 | 3652609.863 | 0.628890287 | 0.662234056 | 0.280657889 |
| LPC 22:0 | 49683.75954 | 55957.59341 | 47709.51999 | 0.802598847 | 0.872144719 | 0.684607517 |
| LPC 22:1 | 5337.651065 | 5960.109427 | 108790.3613 | 0.937872525 | 9.97323E-06 | 7.58811E-06 |
| LPC 22:2 | 23595.29597 | 24361.58616 | 15108.17894 | 0.996769027 | 0.496740484 | 0.371554467 |
| LPC 22:3 | 12992.04382 | 29921.98852 | 23272.01715 | 0.089307137 | 0.317430083 | 0.491409909 |
| LPC 22:4 | 1320151.638 | 1164217.827 | 1127000.365 | 0.566098629 | 0.460784843 | 0.796015563 |
| LPC 22:5 | 2079384.209 | 1538188.36 | 1613960.51 | 0.25856736 | 0.284678321 | 0.983811474 |
| LPC 22:6 | 10690202.25 | 10032565.1 | 10502074.59 | 0.662600537 | 0.650408435 | 0.946920822 |
| LPE 15:0 | 0 | 1812.6175 | 5078.235 | 0.329808115 | 0.16521595 | 0.395850393 |
| LPE 16:1 | 4714.985 | 3265.6175 | 7612.4725 | 0.742743035 | 0.543522193 | 0.340836942 |
| LPE 18:0 | 684254.2348 | 829030.0226 | 725208.1696 | 0.47342405 | 0.882548035 | 0.469809039 |
| LPE 18:1 | 499211.9533 | 497467.622 | 467350.9186 | 0.883140197 | 0.684941192 | 0.736363793 |
| LPE 18:2 | 1578767.006 | 1792825.311 | 1566892.317 | 0.688478196 | 0.806478199 | 0.392994494 |
| LPE 20:3 | 21913.65244 | 52462.39338 | 30056.92287 | 0.017305306 | 0.567104202 | 0.090528914 |
| LPE 20:4 | 751419.1555 | 882769.9127 | 655467.6848 | 0.541654595 | 0.447741896 | 0.106339353 |
| LPE 22:2 | 3259.74 | 1812.6175 | 3261.985 | 0.600464483 | 0.971245748 | 0.578852909 |
| LPE 22:5 | 25928.80621 | 39035.0545 | 25511.46035 | 0.201245194 | 0.962036944 | 0.281433618 |
| LPE 22:6 | 500263.9895 | 528355.2789 | 545405.3857 | 0.917208842 | 0.727531785 | 0.773247007 |
| Biliverdin | 138644.8669 | 103732.0237 | 107301.375 | 0.151171359 | 0.296175434 | 0.732633362 |
| Biliverdin 2 | 87033.75227 | 97112.62382 | 76517.07849 | 0.710989385 | 0.696806597 | 0.451254687 |

IS: ischemic moyamoya disease; HEM: hemorrhagic moyamoya disease; HC: healthy controls; Values shown in the table are mean values unless otherwise noted.

**Table S19: LPC 18:2 level and RNF213 variants in MMD patients in WES**

| RNF213: c.14429G>A | Number of patients | LPC 18:2  Mean peak | Std. Deviation | Std. Error | Levene’s Test for Equality of Variances | t-test for Equality of means |
| --- | --- | --- | --- | --- | --- | --- |
|  |  |  |  |  | Sig. | p value |
| No | 38 (71.7%) | 12722893.84 | 3914179.58 | 634963.77 |  |  |
| Yes | 15 (28.3%) | 11666787.73 | 2600210.96 | 671371.58 |  |  |
| Total | 53 | 12423995.88 | 3598932.55 | 494351.41 | 0.061 | 0.341 |

**Table S20 The result of RNA seq related to LPC (MCA vs STA)**

| Gene Name | Gene Description | log_2_(FC) | P value |
| --- | --- | --- | --- |
| AC136443.1 | phospholipase A2, group X (PLA2G10) pseudogene | -6.72979 | 5.24E-05 |
| PLA2G7 | phospholipase A2 group VII | -6.56013 | 0.00022 |
| PLA2G12AP1 | phospholipase A2 group XIIA pseudogene 1 | -2.77999 | 0.03762 |

**Figure S1: Results of negative ion mode analysis of four subgroups metabolomics**


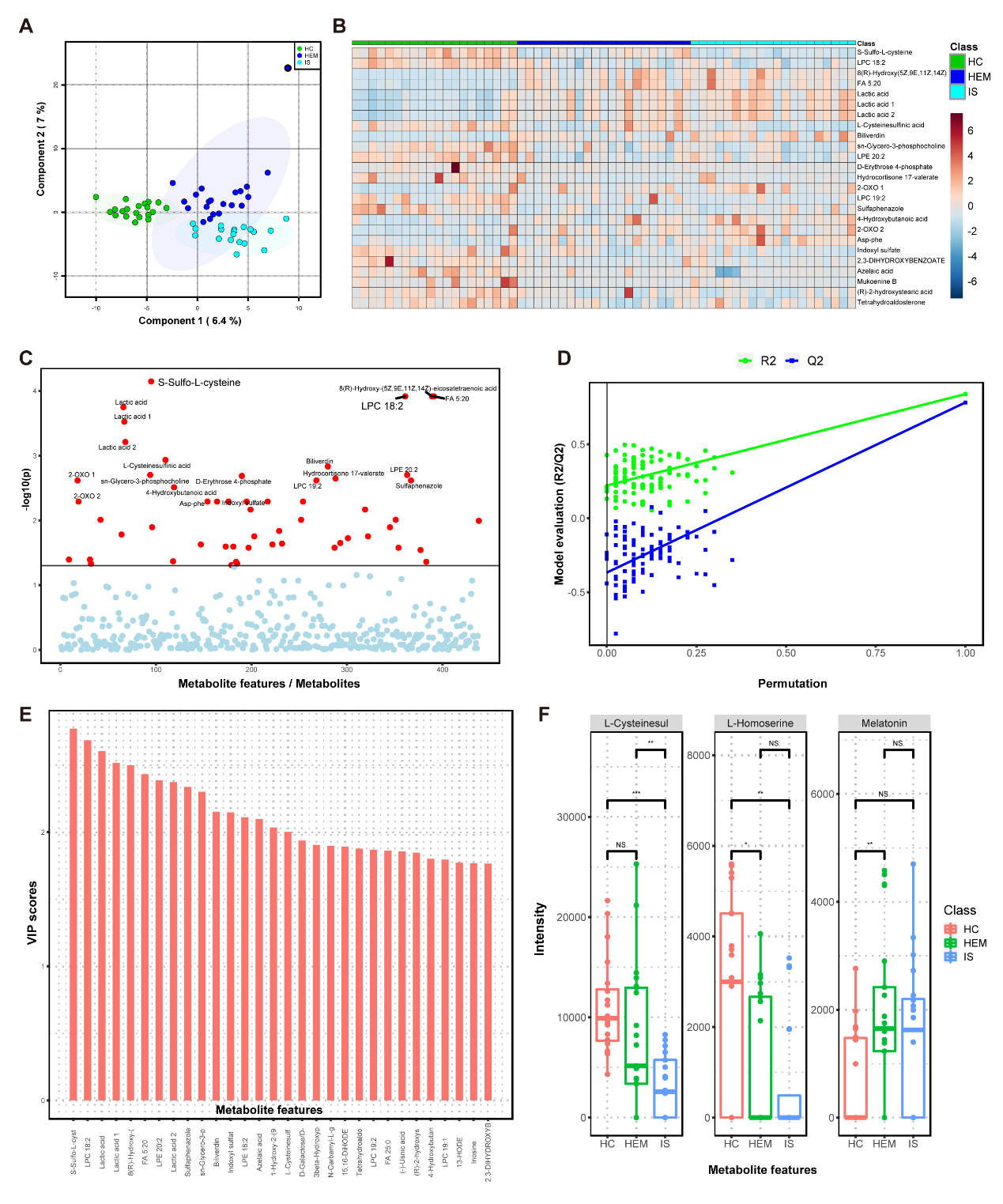


A: PCA results of the four subgroups in positive ion mode. The ellipses of different colors indicate the 95% confidence interval of three subgroups. B: Heat map analysis of three subgroups. The abscissa and the ordinate are the sample and the metabolic mass spectrum characteristics respectively. The color of the dots in the figure represents the intensity of the corresponding sample, corresponding to the characteristics of the metabolic mass spectrum. The graph can directly represent the variation of each feature among samples. The HCA clustering analysis results of sample directions in the figure represent the correlation between samples. C：Graphical expression of mean difference of metabolic mass spectrum characteristics after ANOVA analysis among different categories. The abscissa is the characteristic or metabolite of metabolic mass spectrometry, and the ordinate is the negative logarithmic transformation value of statistical p value. With p=0.05 as the threshold, different colors indicate whether the feature has statistical differences in different categories of samples. The p value is converted by - log10 during mapping, so the differential metabolic feature has a larger Y value in the graph, which is represented by red scatter. D: The result of PLS-DA permutation analysis. The abscissa is the permutation, and the ordinate is the model evaluation (R2/Q2). With model evaluation of change in R2 and Q2 in model, the result showed that hazard of overfitting and availability of the model. E: VIP value result of PLS-DA analysis. The abscissa is the metabolic mass spectrum characteristics, and the ordinate is the VIP value. The figure shows important metabolic mass spectrometry features. VIP>1.0 is the threshold for determining the importance of MS features. F: Box diagram analysis of metabolic mass spectrum characteristics. The abscissa is the characteristic of metabolic mass spectrometry, the samples of different groups are drawn and compared at the same time, and the ordinate is the characteristic intensity, and the analysis results of multiple groups are displayed simultaneously by box diagram analysis. Scatter points in the figure represent the statistical difference between samples and groups, which is represented by the number of points in the upper part of the figure. （***=0.001,**=0.01,*=0.05）

**
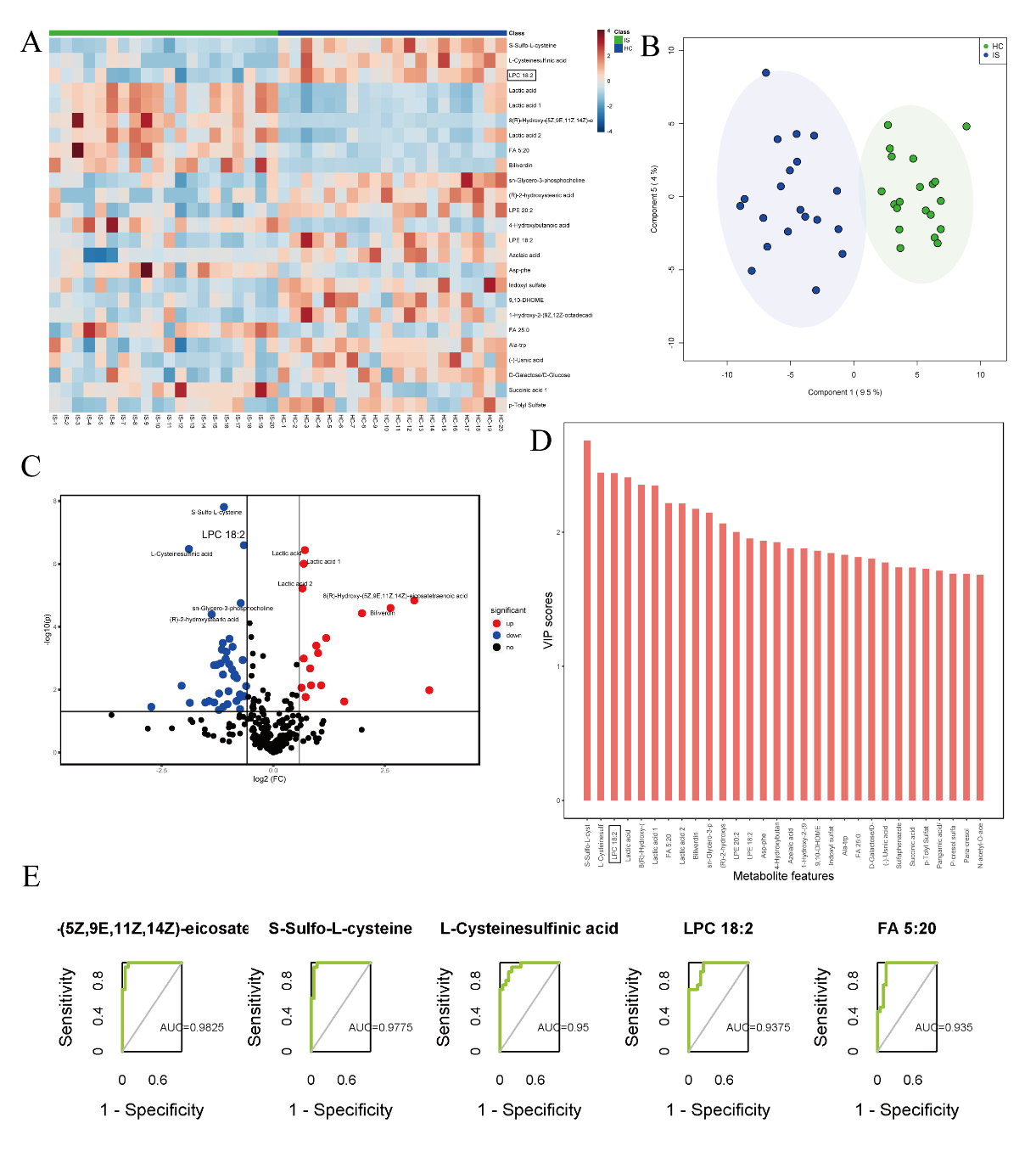
Figure S2: Results of biological analysis of negative ion mode metabolites of in ischemic moyamoya disease (IS) and healthy controls (HC)**

Figure S2 A: Heat map analysis of ischemic moyamoya disease (IS) and healthy controls (HC); B: Score chart of PLS-DA analysis; C: Graphical expression of mean difference of metabolic mass spectrum characteristics; D: VIP value result of PLS-DA analysis; E: The five metabolites with the most significant differences were modeled separately after the ROC curve.

**Figure S3: Three-group ANOVA difference results of LPC16:2/LPC22:1 in targeted metabolomics
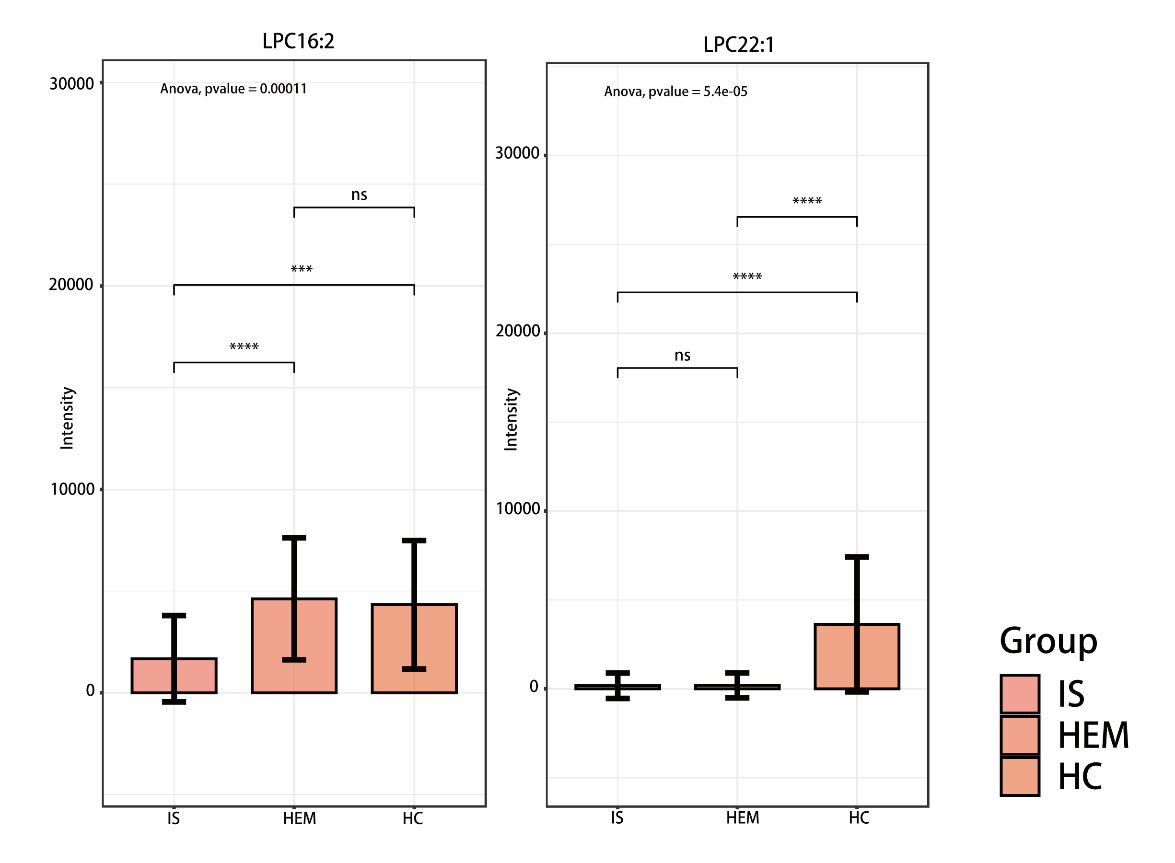
**

Figure S3: Box plot of LPC 16:2 and LPC 22:1 intensity in IS, HEM and HC groups of targeted metabolomics. The left one is LPC 16:2 and three-group differential analysis showed that p value was 0.00011. The right one is LPC 22:1 and p value was 5.4e-05. LPC 16:2 was significantly low in IS group and LPC 22:1 was both low expressed in IS and HEM groups.

**Figure S4. The RNA seq results between MMD and non-MMD diseases.**

**
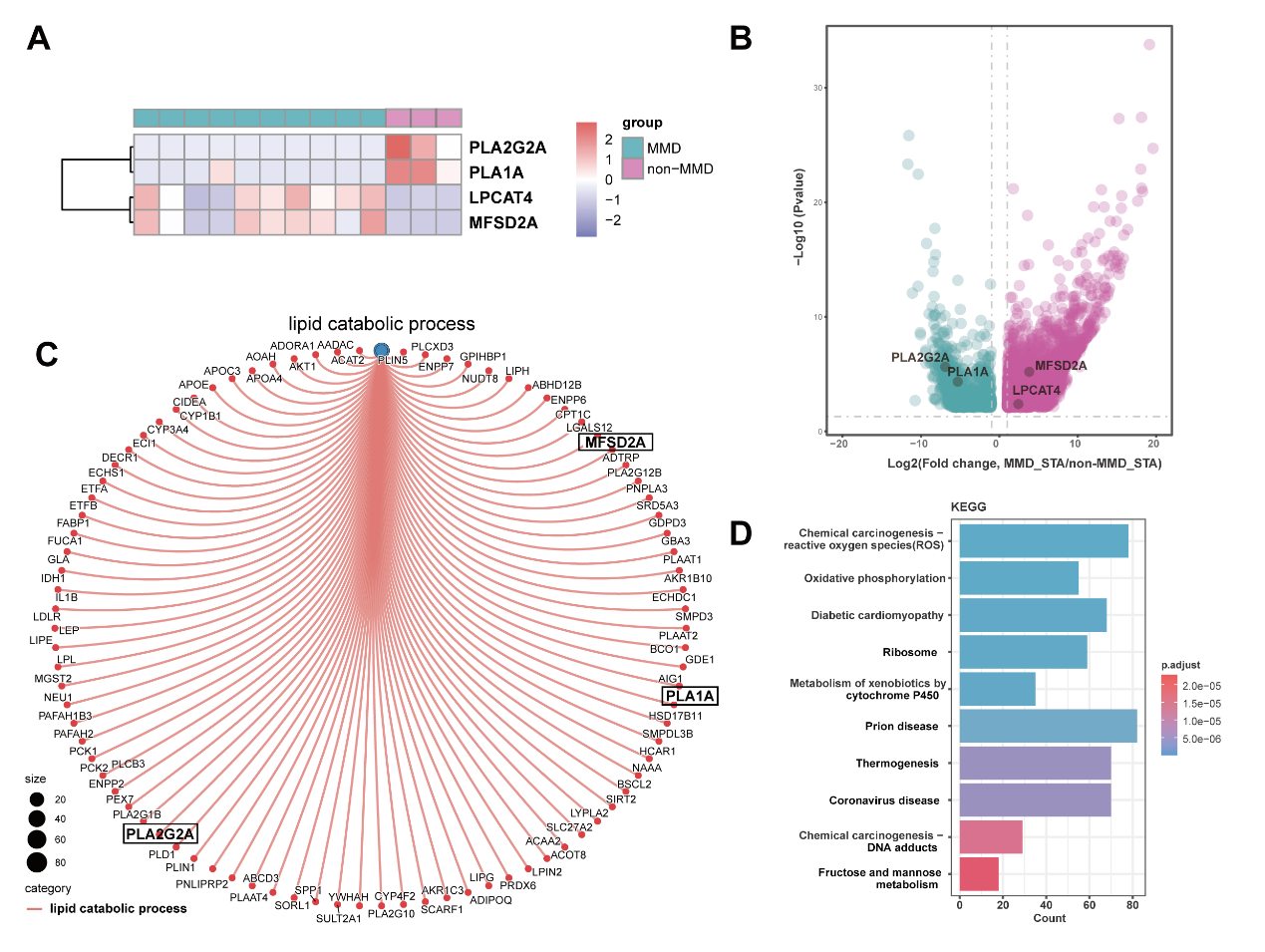
**

A: Heat map of differential expressed genes related with LPC between MMD and non-MMD diseases; B: Volcano map of differential expressed genes related with LCP between MMD and non-MMD diseases; C: Connection between differential expressed genes related with LPC enriched in lipid catabolic process; D: KEGG enrichment pathway analysis of differential expressed genes between MMD and non-MMD diseases.

**Figure S5**. **The ELISA results of different MMD subtypes.**


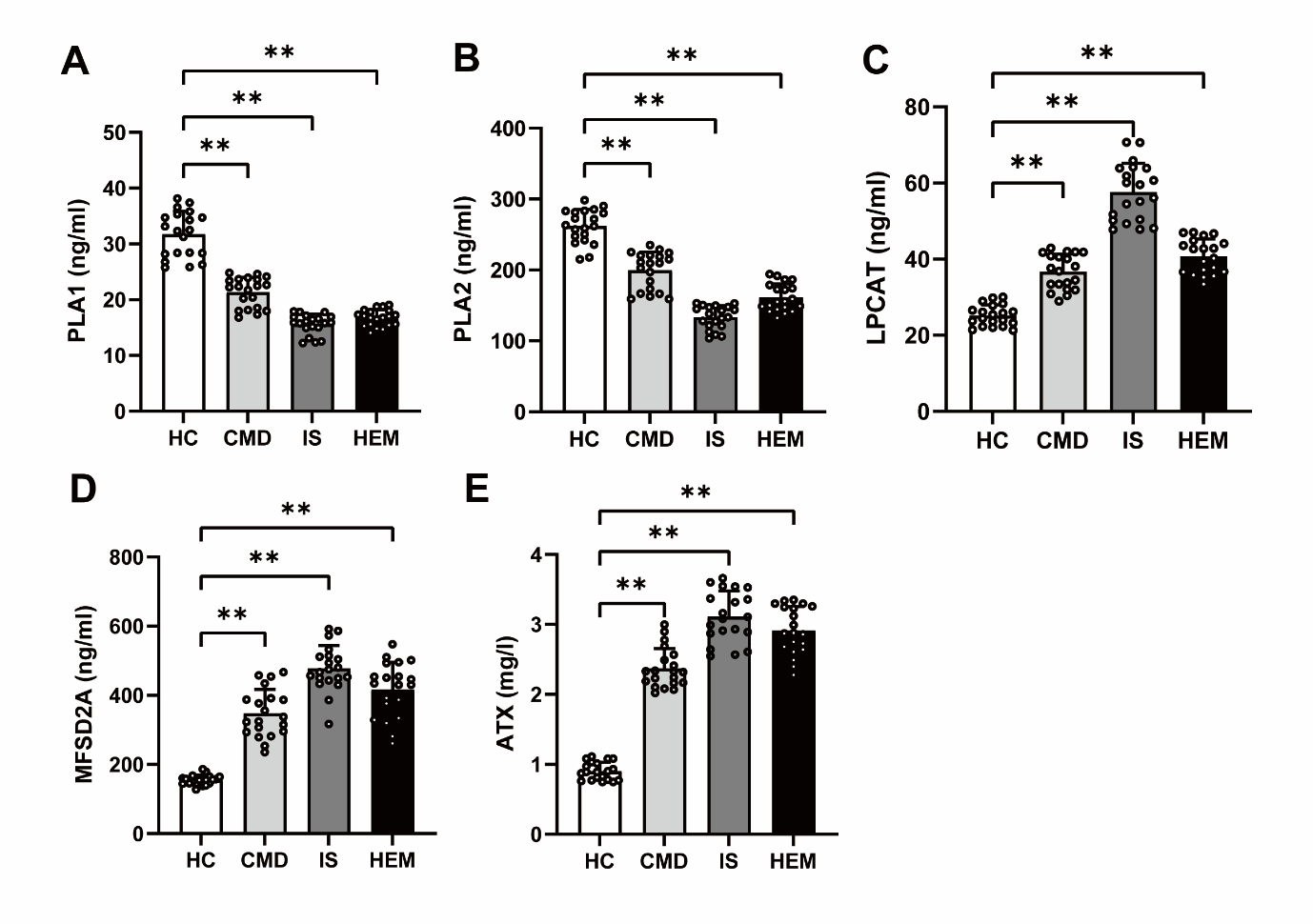


A) PLA1 levels detected in serum from patients with ischemic, hemorrhagic, and pediatric MMD as well as HC using ELISA. B- E) Levels of PLA2 (B), LPCAT (C), Mfsd2a (D) and autotaxin (E) detected in the four subgroups.

**Figure S6. Cell apoptosis, cell viability, MCP-1, NO level and cell cycle analysis of HBVSMCs** **incubated with LPC16 (1, 10, 25, 50 μM) or LPC22 (1, 10, 25, 50 μM) for 24 h.**


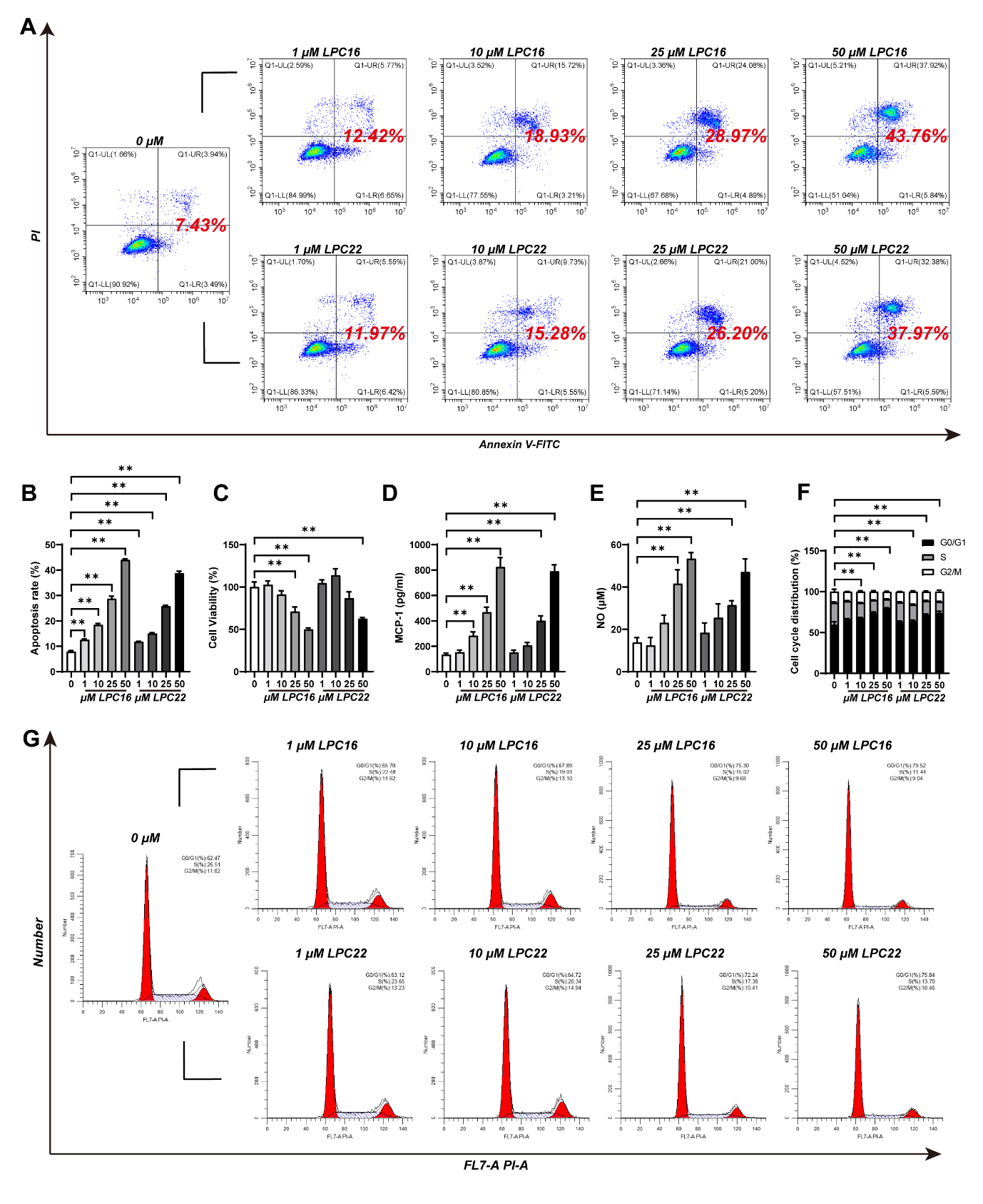


A) Flow cytometry results in 0, 1, 10, 25, 50 μM LPC 16 and LPC 22. The apoptosis ratio of HBVSMCs was positively correlated with the concentration of LPC. B) The apoptosis ratio of HBVSMCs detected by CCK-8 assay after incubation with different concentrations of LPC 16 and LPC 22. C) Cell viability of HBVSMCs incubated in different concentrations of LPC 16 and LPC 22. D) MCP-1 levels measured by ELISA in HBVSMCs incubated with different concentrations of LPC 16 and LPC 22. E) NO expression measured using the NO assay kit in HBVSMCs incubated with different concentrations of LPC 16 and LPC 22. F) Cell cycle distribution of HBVSMCs treated with LPC 16 (1, 10, 25, 50 μM) or LPC 22 (1, 10, 25, 50 μM) for 24 h. G) Number of HBVSMCs in different cell cycle phases after incubation with different concentrations of LPC 16 or LPC 22.

**Figure S7.** **The results of ROS level detection of HBVSMCs and tubule formation assay of HBMECs incubated with LPC 16 (1, 10, 25, 50 μM) or LPC 22 (1, 10, 25, 50 μM) for 24 h.**


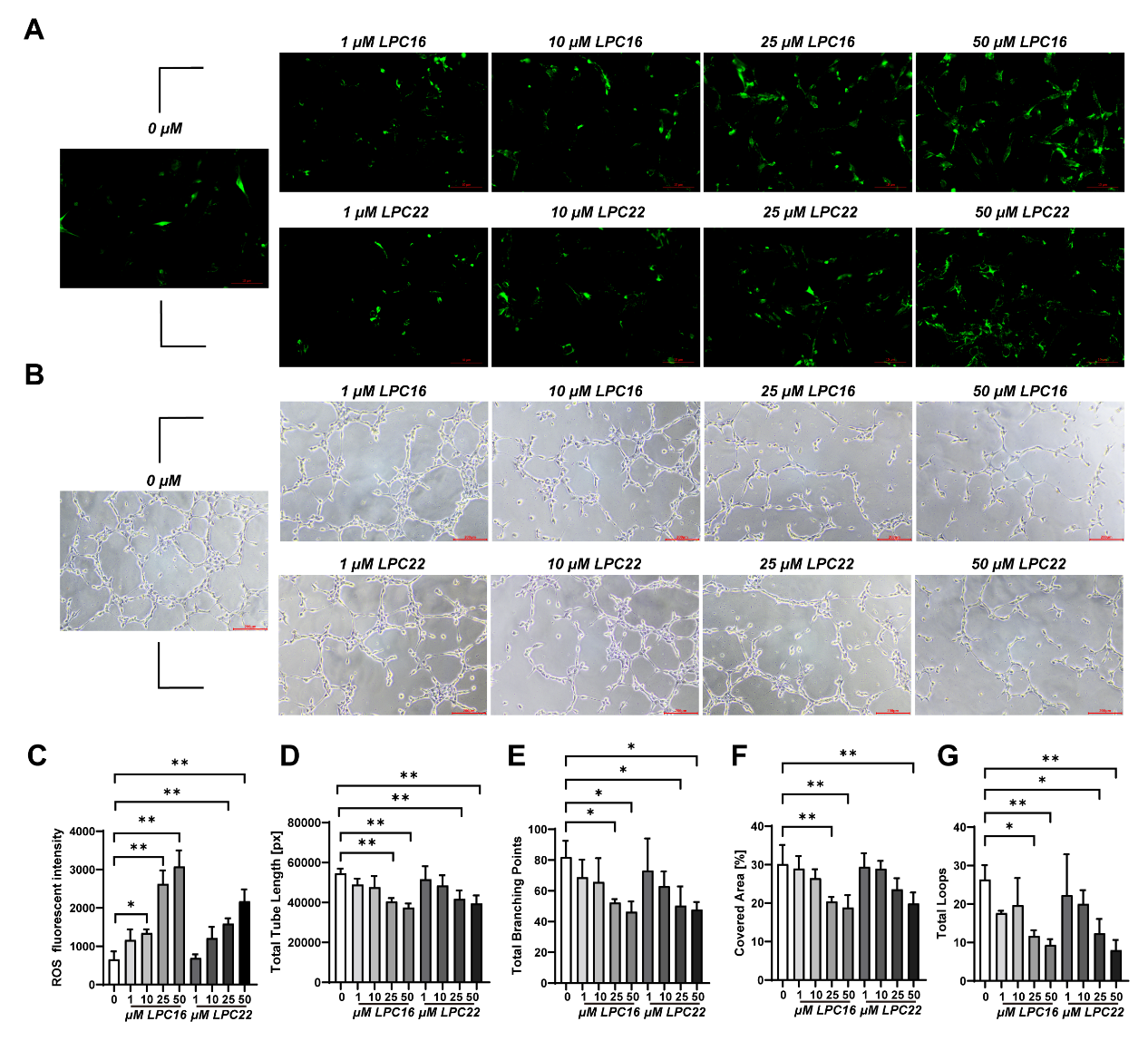


A) The fluorescent pictures of ROS level detection assay of HBVSMCs incubated with different concentrations of LPC. B) The phase contrast pictures of the tubule formation assay of HBMECs incubated with different concentrations of LPC C) ROS production measured by a DCFH-DA probe after HBVSMCs incubation with different concentrations of LPC 16 or LPC 22. 22. D) The total tuber length of HBMECs incubated with different concentrations of LPC 16 or LPC 22. E) The number of total branching points of HBMECs incubated with different concentrations of LPC 16 or LPC 22. serum. F) The percentage of covered area of HBMECs incubated with different concentrations of LPC 16 or LPC 22. G) The number of total loops in the tubule formation assay treated with different concentrations of LPC 16 or LPC 22.

**Figure S8:** **The effect of LPC22 on HBVSMC cells which were incubated with MMD patients’ serum.**


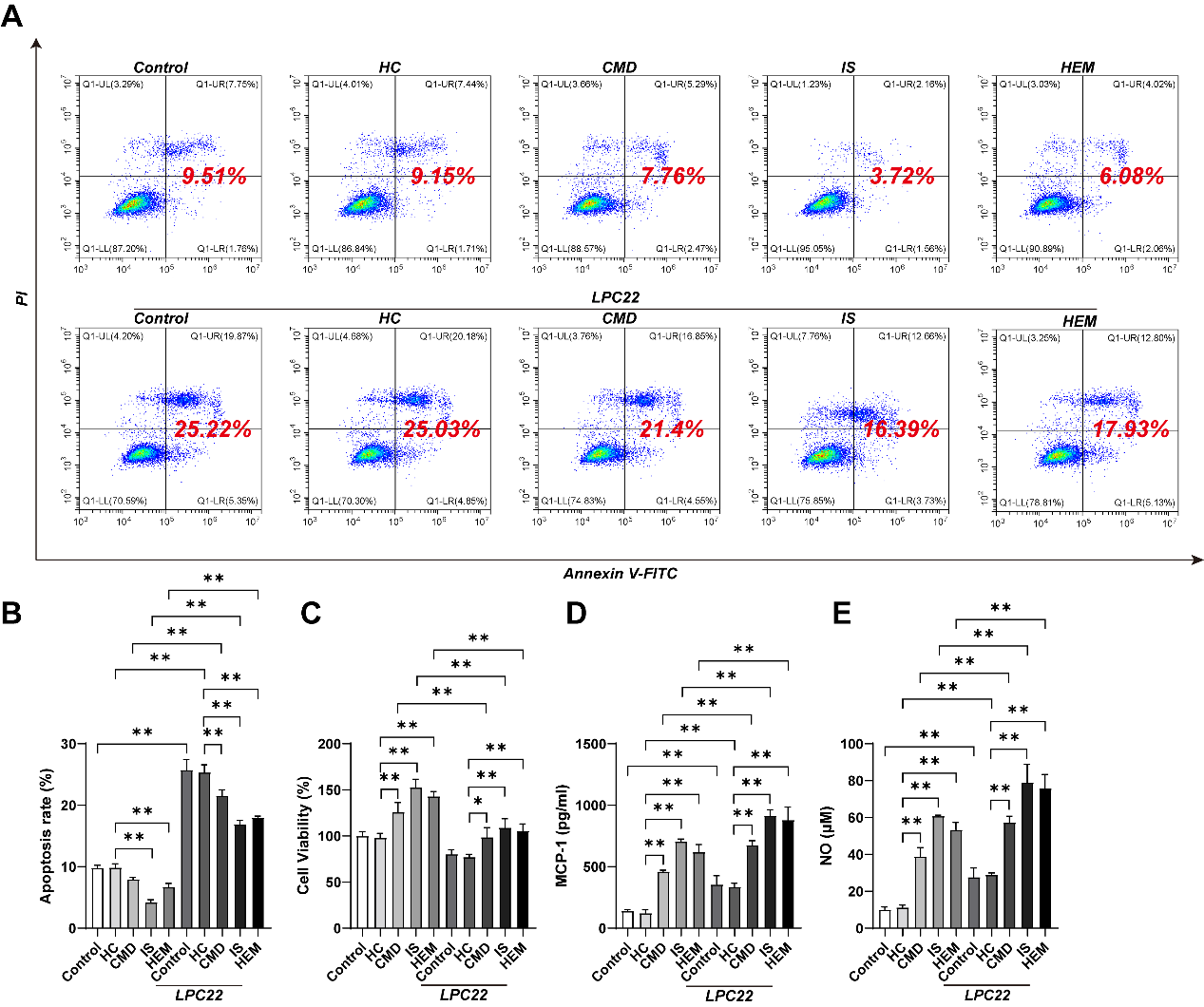


When HBVSMC cells were grown until 80% confluence, they were transferred into serum-reduced cell culture medium with 2% FBS for 24 h, HBVSMC cells were incubated in 2.5% heat-inactivated healthy people’s serum, CMD children’s serum, IS patients’ serum or HEM patients’ serum for 24 h. After that they were cultured with 20 μM LPC22 for 24h. The apoptosis (A, B) ratio of HBVSMC cells in the indicated group was measured by flow cytometric analysis. The cell viability (C) was detected by CCK-8 assay. The levels of MCP-1 (D) were measured by ELISA. The expression of NO (E) was detected by NO assay kit. Results were mean ± SD for three individual experiments. *p<0.05, **p<0.01.

**Figure S9:** **The effect of LPC22 on HBMECs and HBVSMCs which were incubated with MMD patients’ serum.**
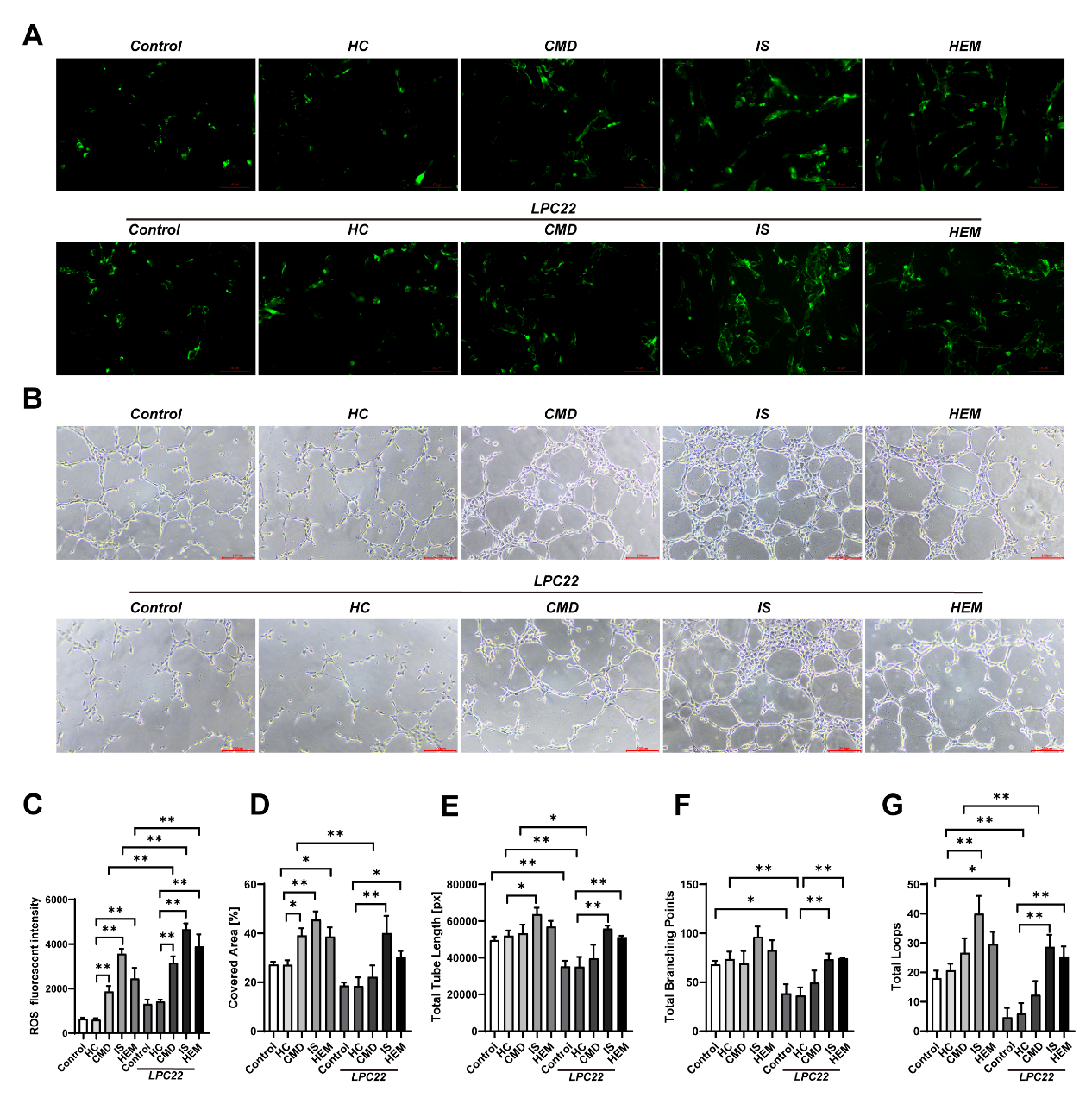


When HBVSMC and HBMEC cells were grown until 80% confluence, they were transferred into serum-reduced cell culture medium with 2% FBS for 24 h, HBVSMC cells were incubated in 2.5% heat-inactivated healthy people’s serum, CMD children’s serum, IS patients’ serum or HEM patients’ serum for 24 h. After that they were cultured with 20 μM LPC22 for 24h. The ROS production in HBVSMC cells were measured by DCFH-DA probe (A, C). The tube formation assay of HBMEC cells (B, D, E, F, G). Results were mean ± SD for three individual experiments. *p<0.05, **p<0.01.

**Figure S10:** **The effect of LPC22 on HBVSMC cells which were incubated with MMD patients’ serum.**
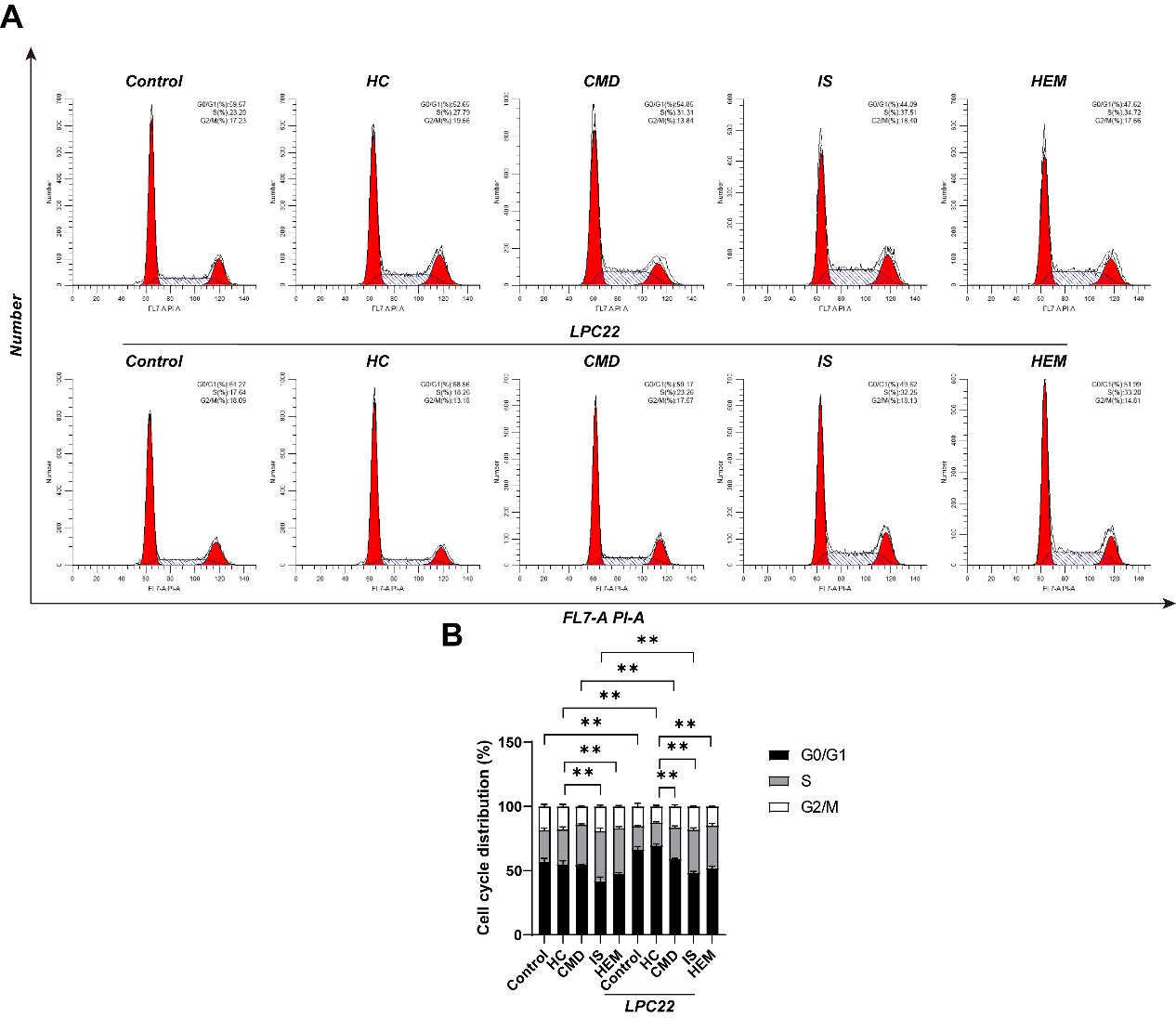


When HBVSMC cells were grown until 80% confluence, they were transferred into serum-reduced cell culture medium with 2% FBS for 24 h, HBVSMC cells were incubated in 2.5% heat-inactivated healthy people’s serum, CMD children’s serum, IS patients’ serum or HEM patients’ serum for 24 h. After that they were cultured with 20 μM LPC22 for 24h. Cell cycle analysis of HBVSMC cells (A, B) cells were performed using flow cytometry. Results were mean ± SD for three individual experiments. *p<0.05, **p<0.01.

**References**

1. Fukui M. Guidelines for the diagnosis and treatment of spontaneous occlusion of the circle of Willis ('moyamoya' disease). Research Committee on Spontaneous Occlusion of the Circle of Willis (Moyamoya Disease) of the Ministry of Health and Welfare, Japan. *Clin Neurol Neurosurg*. 1997;99 Suppl 2:S238-240.

2. Grotta JC. Clinical practice. Carotid stenosis. *N Engl J Med*. 2013;369:1143-1150. doi: 10.1056/NEJMcp1214999

3. Xu S, Wei W, Zhang F, Chen T, Dong L, Shi J, Wu X, Zhang T, Li Z, Zhang J, et al. Transcriptomic Profiling of Intracranial Arteries in Adult Patients With Moyamoya Disease Reveals Novel Insights Into Its Pathogenesis. *Front Mol Neurosci*. 2022;15:881954. doi: 10.3389/fnmol.2022.881954

4. Zheng F, Zhao X, Zeng Z, Wang L, Lv W, Wang Q, Xu G. Development of a plasma pseudotargeted metabolomics method based on ultra-high-performance liquid chromatography-mass spectrometry. *Nat Protoc*. 2020;15:2519-2537. doi: 10.1038/s41596-020-0341-5

5. Wang H, Wang Y, Li X, Deng X, Kong Y, Wang W, Zhou Y. Machine learning of plasma metabolome identifies biomarker panels for metabolic syndrome: findings from the China Suboptimal Health Cohort. *Cardiovasc Diabetol*. 2022;21:288. doi: 10.1186/s12933-022-01716-0

6. Wang Z, Zhou L, Hao W, Liu Y, Xiao X, Shan X, Zhang C, Wei B. Comparative antioxidant activity and untargeted metabolomic analyses of cherry extracts of two Chinese cherry species based on UPLC-QTOF/MS and machine learning algorithms. *Food Res Int*. 2023;171:113059. doi: 10.1016/j.foodres.2023.113059

7. Liu D, Zhao L, Jiang Y, Li L, Guo M, Mu Y, Zhu H. Integrated analysis of plasma and urine reveals unique metabolomic profiles in idiopathic inflammatory myopathies subtypes. *J Cachexia Sarcopenia Muscle*. 2022;13:2456-2472. doi: 10.1002/jcsm.13045

8. Blecharz KG, Frey D, Schenkel T, Prinz V, Bedini G, Krug SM, Czabanka M, Wagner J, Fromm M, Bersano A, et al. Autocrine release of angiopoietin-2 mediates cerebrovascular disintegration in Moyamoya disease. *J Cereb Blood Flow Metab*. 2017;37:1527-1539. doi: 10.1177/0271678x16658301

9. Sugihara M, Morito D, Ainuki S, Hirano Y, Ogino K, Kitamura A, Hirata H, Nagata K. The AAA+ ATPase/ubiquitin ligase mysterin stabilizes cytoplasmic lipid droplets. *J Cell Biol*. 2019;218:949-960. doi: 10.1083/jcb.201712120

10. Piccolis M, Bond LM, Kampmann M, Pulimeno P, Chitraju C, Jayson CBK, Vaites LP, Boland S, Lai ZW, Gabriel KR, et al. Probing the Global Cellular Responses to Lipotoxicity Caused by Saturated Fatty Acids. *Mol Cell*. 2019;74:32-44.e38. doi: 10.1016/j.molcel.2019.01.036

11. Ma W, Cui C, Feng S, Li G, Han G, Hu Y, Li X, Lv J, Liu C, Jin F. Serum Uric Acid and Triglycerides in Chinese Patients with Newly Diagnosed Moyamoya Disease: A Cross-Sectional Study. *Biomed Res Int*. 2019;2019:9792412. doi: 10.1155/2019/9792412

12. Hsieh CC, Yen MH, Liu HW, Lau YT. Lysophosphatidylcholine induces apoptotic and non-apoptotic death in vascular smooth muscle cells: in comparison with oxidized LDL. *Atherosclerosis*. 2000;151:481-491. doi: 10.1016/s0021-9150(00)00453-6

13. Kogure K, Nakashima S, Tsuchie A, Tokumura A, Fukuzawa K. Temporary membrane distortion of vascular smooth muscle cells is responsible for their apoptosis induced by platelet-activating factor-like oxidized phospholipids and their degradation product, lysophosphatidylcholine. *Chem Phys Lipids*. 2003;126:29-38. doi: 10.1016/s0009-3084(03)00091-4
